# Genotypic variation in winter wheat for fusarium foot rot and its biocontrol using *Clonostachys rosea*

**DOI:** 10.1093/g3journal/jkae240

**Published:** 2024-10-07

**Authors:** Sidhant Chaudhary, Rosa Margarida Nogueira Ricardo, Mukesh Dubey, Dan Funck Jensen, Laura Grenville-Briggs, Magnus Karlsson

**Affiliations:** Department of Forest Mycology and Plant Pathology, Swedish University of Agricultural Sciences, Uppsala SE-75007, Sweden; Department of Forest Mycology and Plant Pathology, Swedish University of Agricultural Sciences, Uppsala SE-75007, Sweden; Department of Forest Mycology and Plant Pathology, Swedish University of Agricultural Sciences, Uppsala SE-75007, Sweden; Department of Forest Mycology and Plant Pathology, Swedish University of Agricultural Sciences, Uppsala SE-75007, Sweden; Department of Plant Protection Biology, Swedish University of Agricultural Sciences, Lomma SE-23422, Sweden; Department of Forest Mycology and Plant Pathology, Swedish University of Agricultural Sciences, Uppsala SE-75007, Sweden

**Keywords:** biological control, *Clonostachys rosea*, disease resistance, *Fusarium graminearum*, GWAS, wheat, Plant Genetics and Genomics

## Abstract

Biological control to manage plant diseases is an environmentally friendly alternative to using chemical pesticides. However, little is known about the role of genetic variation in plants affecting the efficacy of biological control agents (BCAs). The aim of this study was to explore the genetic variation in winter wheat for disease susceptibility to fusarium foot rot caused by *Fusarium graminearum* and variation in biocontrol efficacy of the fungal BCA *Clonostachys rosea* to control the disease. In total, 190 winter wheat genotypes were evaluated under controlled conditions in 2 treatments, i.e. (1) *F. graminearum* (Fg) and (2) *F. graminearum* infection on *C. rosea*–treated seeds (FgCr). Alongside disease severity, plant growth-related traits such as shoot length and root length were also measured. Comparison of genotypes between the 2 treatments enabled the dissection of genotypic variation for disease resistance and *C. rosea* efficacy. The study revealed significant variation among plant genotypes for fusarium foot rot susceptibility and other growth traits in treatment Fg. Moreover, significant variation in *C. rosea* efficacy was also observed in genotype contrasts between the 2 treatments for all traits. Using a 20K marker array, a genome-wide association study was also performed. We identified a total of 18 significant marker–trait associations for disease resistance and *C. rosea* efficacy for all the traits. Moreover, the markers associated with disease resistance and *C. rosea* efficacy were not co-localized, highlighting the independent inheritance of these traits, which can facilitate simultaneous selection for cultivar improvement.

## Teaser

This study explored genetic variation in winter wheat for disease resistance and the biocontrol efficacy of a beneficial fungus, Clonostachys rosea, in controlling *Fusarium graminearum* causing fusarium foot rot. Testing 190 wheat genotypes, significant variation in disease resistance and biocontrol efficacy was found. Further genome-wide analysis revealed independent inheritance of disease resistance and biocontrol efficacy, suggesting a possibility for simultaneous breeding for these traits. It can contribute to sustainable agriculture practices by reducing chemical pesticide use and enhancing biocontrol effectiveness.

## Introduction

Agricultural production relies heavily on the use of chemical pesticides to achieve optimal yields and quality. According to the latest report from the Food and Agriculture Organization of the United Nations (FAO 2022), pesticide usage has increased by about 50% from 1.2 kg/ha in 1990 to 1.8 kg/ha in 2020 with a total amount of active ingredients at 2.7 million tons. The overreliance of agricultural systems on chemical pesticides has led to negative environmental impacts such as soil and water contamination, impacting nontargeted plants and animals, and biodiversity losses (Tudi *et al*. 2021). Moreover, resistance evolution to pesticide application in pathogens is a severe problem affecting efficacy and future crop security (Gould *et al*. 2018; Karlsson Green *et al*. 2020). Integrated pest management (IPM) approaches to managing pests and pathogens below economic injury levels using a combination of sustainable methods offers considerable potential to reduce the dependence on chemical pesticides in agricultural systems. Furthermore, the European Union Framework Directive 2009/128/EC asks all plant production professionals to comply with IPM principles (European Union 2009; Barzman *et al*. 2015; Karlsson Green *et al*. 2020). One such potential IPM approach is using biological control methods for pest and pathogen management. The use of biological control is specifically recommended in the European Commission's proposal for a new regulation on the sustainable use of plant protection products to reduce the use of synthetic chemical pesticides by 50% by 2030 as per the European Green Deal (European Commission 2022).

Biological control, or biocontrol, is defined as the exploitation of living organisms (biological control agents, BCA) to combat pests and pathogens, directly or indirectly, to provide human benefits (Stenberg *et al*. 2021). There are already numerous bacterial, fungal, oomycete, and viral BCAs that have been isolated, tested, and successfully commercialized (Collinge *et al*. 2022). The global market for BCAs is continuously growing, with a market value of 5.61 billion USD in 2021 and with a projected market value in 2029 of 18.15 billion USD in 2029, reflecting the demand from various players involved in plant protection (Fortune Business Insights 2022). The modes of action of BCAs can be classified into 4 categories: (1) exploitative competition for resources such as oxygen, carbon, nitrogen, and other vital nutrients, (2) interference competition for space, achieved through antibiosis, where the BCA inhibits the pathogen by producing toxic specialized metabolites or enzymes, (3) hyperparasitism, where the BCA acts as a predator, preying on the pathogen, (4) induced resistance, involving the indirect interaction of a BCA by triggering plant defense mechanisms against invading pathogens (Jensen *et al*. 2017; Collinge *et al*. 2022). It is possible for a BCA to exhibit more than one mode of action against a pathogen and it can vary depending on the pathogen, plant, and other environmental factors (Jensen *et al*. 2021).

*Clonostachys rosea* is one such BCA, which is an ascomycete fungus with a generalist lifestyle including saprotrophism, plant endophytism, and mycoparasitism (Schroers *et al*. 1999; Jensen *et al*. 2021). Using *C. rosea* in augmentative biological control strategies, where it is released into target areas after mass-rearing, it has been reported to exhibit biocontrol properties against a multitude of fungal and oomycete pathogens. Different strategies employed by *C. rosea* in interactions with other microorganisms, such as competition for nutrients and space (Sutton *et al*. 1997), antibiosis (Han *et al*. 2020; Saraiva *et al*. 2020), induction of plant defense responses (Wang *et al*. 2019; Kamou *et al*. 2020), and direct parasitism (Barnett and Lilly 1962; Jensen *et al*. 2021), are reported in the literature. *C. rosea* strain IK726 was isolated from barley roots in 1992 (Knudsen *et al*. 1995), the genome was sequenced in 2015 (Karlsson *et al*. 2015), and it has been explored in detail for its mycoparasitism and modes of action. As summarized in Jensen *et al*. (2021), *C. rosea*–mediated biocontrol is observed against a multitude of pathogens, such as *Botrytis cinerea* in strawberry, raspberry, rose, and tomato; *Fusarium* spp. in tomato, pine, cereals, and pulses; *Plasmodiophora brassicae* in Brassicaceae crops; *Puccinia* spp. in cereals; *Zymoseptoria tritici* in wheat; *Alternaria* spp. in tomato, carrot, and pulses; *Pythium* spp.; and *Phytophthora* spp. in various crops.

Plant breeding is another integral part of sustainable agriculture and IPM, offering a sustainable and cost-effective approach to pest control by enhancing resistance to biotic and abiotic stresses and increasing yield. Breeding efforts for winter wheat in Europe in the last decades have led to a steady increase in yield potential and improved resistance to diseases and abiotic stresses (Voss-Fels *et al*. 2019; Leišová-Svobodová *et al*. 2020; Zetzsche *et al*. 2020; Laidig *et al*. 2021). Among the pathogens in wheat cultivation, *Fusarium* spp., which are often present as a species complex, are one of the most devastating and economically important groups of pathogens infecting various plant parts at different growth stages, causing fusarium foot rot, fusarium root rot, fusarium seedling blight, fusarium crown rot, and fusarium head blight (Dean *et al*. 2012; Karlsson *et al*. 2021). *Fusarium graminearum*, *Fusarium culmorum*, *Fusarium avenaceum*, and *Fusarium poae* are the species with the highest incidence of fusarium head blight in Europe (Becher *et al*. 2013). In the last decades, a lot of breeding efforts have been made to identify quantitative trait loci (QTL) for the management of fusarium head blight across the globe (Buerstmayr *et al*. 2020). In addition to causing fusarium head blight, *Fusarium* spp. are also economically important pathogens causing ground-level and below-ground diseases in dry climates across continents (Kazan and Gardiner 2018). Moreover, with the changing climate and increasing temperatures in northern Europe, *F. graminearum* is also predicted to become more important in the future (Strandberg *et al*. 2024). While the understanding of *F. graminearum* causing head blight is well-developed, knowledge about its infestation at early stages, leading to blights, foot rot, and root rot, remains limited (Voss-Fels *et al*. 2018). Furthermore, resistance to fusarium head blight does not always correlate with resistance to fusarium crown rot and fusarium root rot, which is suggested to be due to differences in host plant resistance (Li *et al*. 2010; Wang *et al*. 2015). Therefore, it is essential to explore the genetic architecture for resistance to *F. graminearum* causing ground-level and below-ground diseases.

Alongside resistance genotypes, chemical seed treatment is used to manage seed-borne and seedling-stage diseases. Seed treatment with BCAs, instead of chemical pesticides, can be an environmentally friendly alternative (Jensen *et al*. 2000). However, it has been frequently proposed that the disease control efficacy of BCAs can be modulated by plant genotype variation (Smith and Goodman 1999; Stenberg *et al*. 2015; Köhl *et al*. 2019; Collinge *et al*. 2022). However, these tri-partite interactions among plant genotypes–pathogen–BCA have mostly been explored with a limited number of plant genotypes. Moraga-Suazo *et al*. (2016) reported a differential response of 2 contrasting *Pinus radiata* genotypes toward *C. rosea*–mediated biocontrol of the pitch canker pathogen *Fusarium circinatum*. The study demonstrated the ability of *C. rosea* to produce plant genotype-specific induced systemic resistance (ISR). Tucci *et al*. (2011) also reported differences among 5 tomato genotypes for enhanced ISR against the gray mold pathogen *B. cinerea* using *Trichoderma atroviride* and *Trichoderma harzianum.* Furthermore, Arkhipov *et al*. (2023) showed variation among 6 tomato genotypes toward *Phytophthora capsici* biocontrol by *Pseudomonas azotoformans*, which involved induction of ISR involving a hypersensitive response. Ryan *et al*. (2004) reported differences in the effectiveness of BCAs for potato scab among 5 cultivars in field trials. Biocontrol efficacy of *Pseudomonas fluorescens* against *Pythium ultimum* was also observed to differ among 3 wheat cultivars (Meyer *et al*. 2010). Furthermore, Rebeka *et al*. (2013) revealed significant differences among 50 genotypes for *Fusarium oxysporum* compatibility in controlling *Striga hermonthica*. In a study by Smith *et al*. (1999), variation among 61 tomato genotypes in interacting with disease suppressive bacteria *Bacillus cereus* is shown against the pathogen *Pythium torulosum*. Moreover, differences among plant genotypes were also observed for biostimulation by *Trichoderma* spp. as shown in sugar beet for plant dry weight and shoot dry weight (Schmidt *et al*. 2020) and lentils for root and shoot development parameters (Prashar and Vandenberg 2017). These examples show that plant genotypes impact the compatibility between plants and beneficial microorganisms. Therefore, considering plant genetic variation is crucial for the effective deployment of BCAs. Understanding the genetic basis of host plant interactions with BCAs offers opportunities to augment traditional plant breeding for yield and resistance traits with enhanced compatibility with beneficial microorganisms.

In this study, we hypothesized that wheat genotypes vary in their susceptibility to *F. graminearum* causing foot and root rot and *C. rosea*–mediated biocontrol efficacy to control the disease. Specifically, the objectives were to (1) test for plant genotype variation in 190 winter wheat genotypes for resistance to *F. graminearum* causing foot and root rot; (2) test for plant genotype variation for *C. rosea*–induced biocontrol efficacy against fusarium foot and root rot; and (3) conduct a genome-wide association study (GWAS) to identify marker–trait associations of fusarium foot and root rot disease resistance and *C. rosea*–mediated biocontrol efficacy, and to determine whether these traits are inherited together or independently.

## Materials and methods

### Plant and fungal material

In this study, a total of 190 winter wheat genotypes were used, which included landraces and cultivars initially obtained from the Nordic Genetic Resource Center and later multiplied (Supplementary Table 1). For foot and root rot disease, *F. graminearum* strain PH1 was used as the pathogen in this study (Trail and Common 2000). The strain was revived from −80°C glycerol stock and grown on potato dextrose agar (PDA) media (BD Difco Laboratories, France) at 20°C in dark conditions. BCA *C. rosea* strain IK726, initially isolated from barley roots in Denmark, was used (Knudsen *et al*. 1995). The strain was revived from a glycerol conidial stock stored at −80°C and grown on PDA media petri plates at 20°C in dark conditions.

### Bioassay setup

Bioassays for *F. graminearum* foot and root rot and *C. rosea* biocontrol efficacy were conducted in the sand seedling test modified from the test described previously (Knudsen *et al*. 1995). In total, surface sterilized seeds of 190 genotypes were tested for FRR disease resistance and *C. rosea* biocontrol efficacy under 2 treatments: (1) Fg (pathogen only) and (2) FgCr (pathogen and BCA *C. rosea*). Three seeds were sown per pot (5 × 5 × 5 cm) in trays of 40 pots. Pathogen inoculation was carried out in both treatments by placing a 5 mm diameter *F. graminearum* agar plug equidistant from seeds in the pot. For the BCA seed coating in the treatment FgCr, a conidial suspension of *C. rosea* was made by flooding the PDA plates with sterile water, followed by filtration through Miracloth (Merck KGaA, Darmstadt, Germany) to remove mycelia and growth media. Seed surface coating with *C. rosea* conidia at the concentration of 1 × 10^6^ cfu/mL (colony forming units per mL) was performed by shaking the seeds in *C. rosea* suspension on a rotary shaker at 120 rpm for 30 min. For treatment Fg, seeds were shaken as above in sterile water.

To accommodate 190 winter wheat genotypes, the experiment was conducted in 6 batches, each testing a subset of genotypes. Within each batch, a randomized complete block design was used with 5 trays randomly assigned to each treatment (Fg and FgCr), making 5 biological replicates per genotype. To account for batch-to-batch variation, 3 check genotypes (Kranich, Stava, and Festival) were used in all trays of each batch. Trays were kept in a growth chamber with a photoperiod of 16 h light (200 μmol/m^2^ s) at 20°C and 8 h dark at 16°C. Plants were grown for 19 days and the germinated seedlings were harvested and evaluated for disease symptom scoring on a 0–4 scale with 0.5 increments, 0 = healthy plants with no symptoms and 4 = dead plants, as previously described (Knudsen *et al*. 1995). Moreover, shoot and root length (±0.5 cm) were measured and combined to make plant length (±1 cm).

### Phenotypic data analysis

Unadjusted arithmetic means from each pot were used for the analysis. To estimate the best linear unbiased estimators (BLUEs) of genotypes in treatments Fg and FgCr, a mixed model approach using Kenward–Roger's approximation of the degrees of freedom was used (Kenward and Roger 1997). The model is as follows:


$$y_{ijkl}\;=\;\mu \;+\;r_{i}\;+\;b_{ij}\;+\;g_{k}\;+\;t_{l}\;+\;(gt{)}_{kl}\;+\;\varepsilon_{ijkl}$$

where $y_{ijkl}$ is the BLUE estimate for the *y*-th trait of the *k-*th genotype in the *l-*th treatment, μ denotes the overall mean; $r_{i}$ is the effect of the *i-*th batch, $b_{ij}$ the effect of the *j-*th block nested within the *i-*th batch, $g_{k}$ the effect of the *k-*th genotype, $t_{l}$ the effect of *l-*th treatment, $(gt{)}_{kl}$ the interaction effect of the *k-*th genotype with the *l-*th treatment, and $\varepsilon_{ijkl}$ the residual term. Batches and blocks nested within batches were treated as random factors.

Analysis of variance (ANOVA) was performed on the model to evaluate the significance of various model terms. BLUEs were estimated for genotypes in each treatment. Inter–treatment contrasts for each genotype were used as estimators for the biocontrol efficacy effect. To facilitate interpretation, the contrast direction was Fg–FgCr for disease score, where a positive value indicated disease reduction in *C. rosea* seed treatment; while the contrast direction was FgCr–Fg for shoot length, root length, and plant length, where a positive value indicated length increase with *C. rosea* seed treatment. A post–hoc Tukey's test was performed to test the significance of inter–treatment contrasts, and false discovery rate–adjusted *P*-values were used to correct for multiple testing (Benjamini and Hochberg 1995). Broad-sense heritability of traits as $H_{P}^{2}$ after Piepho and Möhring (2007) and $H_{C}^{2}$ after Cullis *et al*. (2006) was also estimated separately in each treatment following a reduced version of the above-described model without any treatment effect and genotype × treatment interaction effect.

### Genome-wide association analysis

A total of 181 out of 190 winter wheat genotypes used in the current panel were previously genotyped using a 20K single nucleotide polymorphism (SNP) marker array at TraitGenetics GmbH, Germany (Odilbekov *et al*. 2019). A total of 7,360 SNP markers were retained for the GWAS after filtering out the markers with >20% missing alleles and <5% minor allele frequency. The remaining missing alleles were imputed to the major allele. For GWAS, a total of 5 different models were used as follows: GLM (Price *et al*. 2006), MLM (Yu *et al*. 2006), MLMM (Segura *et al*. 2012), FarmCPU (Liu *et al*. 2016), and BLINK (Huang *et al*. 2019). GLM and MLM are single-locus GWAS models, whereas MLMM, FarmCPU, and BLINK are multiple loci models. To correct for relatedness and population structure, the kinship matrix and the first 17 principal components (explaining 50% variation) were used as covariates in the analyses. For significant marker–trait association, a threshold of negative log (1/number of SNP markers) was used to overcome the over stringency of the Bonferroni test threshold (0.05/number of SNP markers) and low sample size (Yang *et al*. 2011; Wang *et al*. 2012). For each significant marker at the negative log threshold, an allelic level comparison was made for the phenotypic distribution of the trait using 1-way ANOVA, followed by a Tukey's post–hoc test. Heterozygous alleles with a frequency <5 were dropped prior to the comparisons.

All statistical analyses were performed using the statistical software R version 4.3.1 “Beagle Scouts” (R Core Team 2023). The linear mixed model analysis was performed using the package “lme4” version 1.1-35.3 (Bates *et al*. 2015) and its extension “lmerTest” version 3.1-3 (Kuznetsova *et al*. 2017). In addition, the estimation of BLUEs and post–hoc comparisons of individual genotypes between treatments were performed using packages “emmeans” version 1.10.1 (Lenth 2023), “multcomp” version 1.4-25 (Hothorn *et al*. 2008), and “multcompView” version 0.1-10 (Graves *et al.* 2023). Genome-wide association analysis was performed using the genome association and prediction integrated tool (GAPIT) version 3 (Wang and Zhang 2021). “Tidyverse” suite version 2.0.0 was used for most data processing and visualization alongside other dependency packages (Wickham *et al*. 2019).

### Candidate gene identification

To search for genes localized at significant SNP marker–trait associations, a stringent window of ± 100 kb was explored. Firstly, the physical positions of SNP markers were identified by mapping SNP marker sequences against the *Triticum aestivum* IWGSC CS RefSeq v2.1 genome (GCF_018294505.1) using the BLAST algorithm (Altschul *et al*. 1990). Genes localized within ± 100 kb surrounding significant SNP markers were filtered using the gene annotation data available at the National Center for Biotechnology Information (NCBI) *T. aestivum* release 100 (2021 October 27th). Further description of the genes was performed by searching the filtered genes in the gene library at NCBI.

## Results

### Performance of wheat genotypes across treatments

**Fig. 1.**
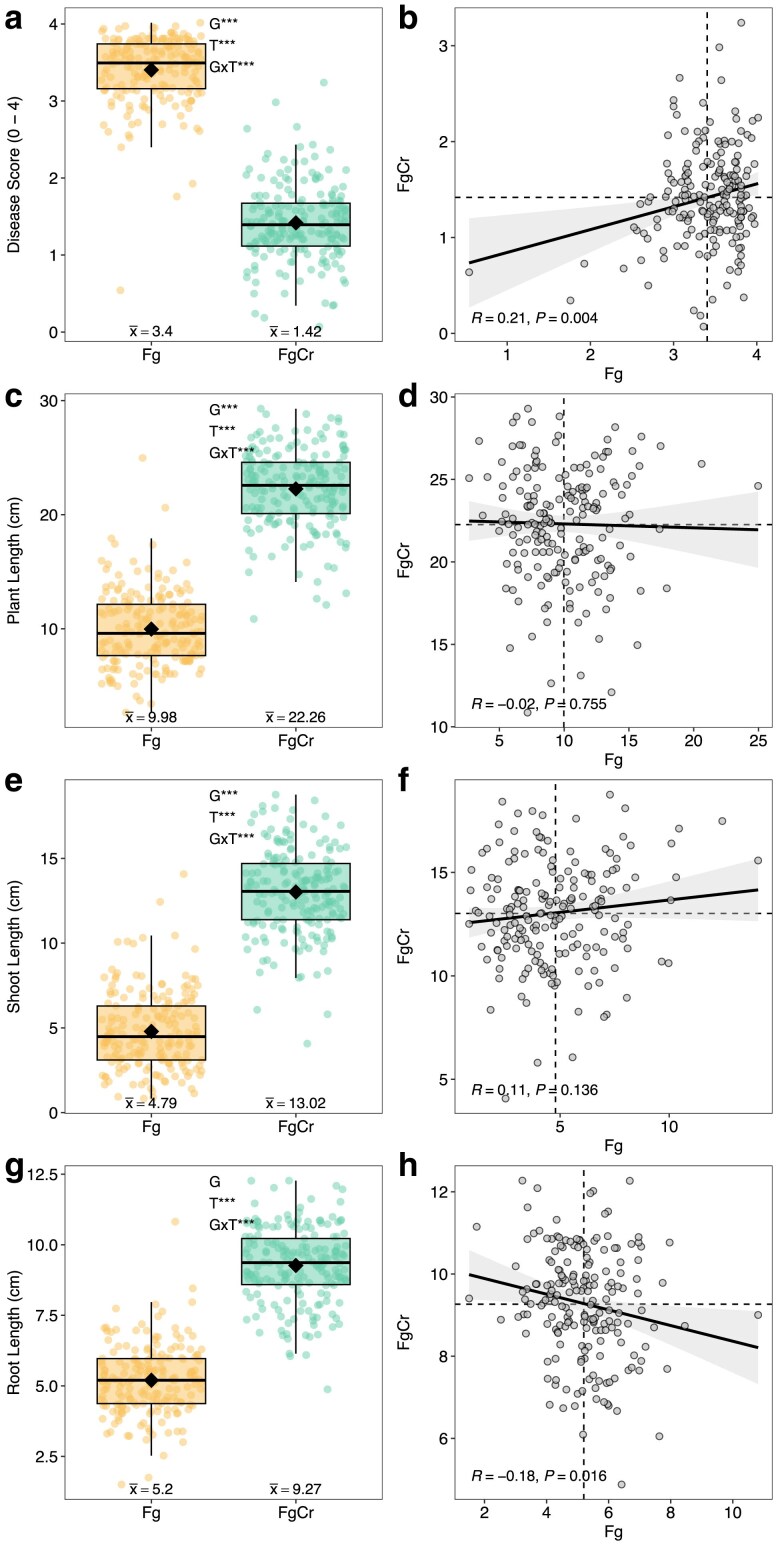
Comparisons and correlations between 2 treatments Fg (*Fusarium graminearum*) and FgCr (*F. graminearum* and *Clonostachys rosea* seed treatment). Box plots show comparison of BLUEs of genotypes in treatments Fg and FgCr for disease score (a), plant length (c), shoot length (e), and root length (g). Thick horizontal line in the box represents the median and black diamond represents the mean estimate of each treatment. G, T, and G × T annotation summarize the ANOVA results for genotype effect, treatment effect, and genotype-by-treatment interaction effect, respectively. ***Significance at *P* < 0.001. Pearson's correlation coefficient between treatment Fg and FgCr are shown for disease score (b), plant length (d), shoot length (f), and root length (h). Dashed vertical and horizontal lines indicate the mean estimate of the trait in treatment Fg and FgCr, respectively.

The performance of 190 winter wheat genotypes for fusarium foot rot and its biocontrol by *C. rosea* was evaluated in the absence (Fg) and presence (FgCr) of *C. rosea* seed treatment. Significant differences (*P* < 0.001) between treatments were observed for disease score, plant length, shoot length, and root length (Table 1). On average, the disease score was reduced by approximately half in treatment FgCr (1.42 ± 0.5) in comparison to treatment Fg where the disease was very high (3.4 ± 0.44) (Fig. 1a, Table 2, Supplementary Table 2). Similarly, estimates for root length, shoot length, and total plant length were almost doubled in treatment FgCr with *C. rosea* seed treatment (Fig. 1c, e, and g, Table 2, Supplementary Table 2). Heritability estimates for all traits ranged from low to moderate ranging from 0.14 to 0.6 for $H_{P}^{2}$ and from 0.11 to 0.51 for $H_{C}^{2}$. Heritability estimates were lower in treatment Fg than in FgCr for disease score and shoot length, similar across treatments for plant height, and higher in treatment Fg than in FgCr for root length (Table 2). Overall, the 4 traits used in this study were found in highly significant correlation (*R* > |0.85|, *P* < 0.001) among each other (Supplementary Fig. 1). The 3 growth-related traits plant length, shoot length, and root length were in strong positive correlation with each other. Disease score was in the overall strong negative correlation with plant length (*R* = −0.92, *P* < 0.001), shoot length (*R* = −0.91, *P* < 0.001), and root length (*R* = −0.87, *P* < 0.001), emphasizing the impact of disease severity on growth. Particularly, a negative correlation of disease score was weaker in treatment FgCr for plant length (*R* = −0.44, *P* < 0.001), shoot length (*R* = −0.43, *P* < 0.001), and root length (*R* = −0.31, *P* < 0.001), suggesting a variable effect of *C. rosea* in reducing fusarium foot rot among wheat genotypes along with a variable impact on plant growth (Supplementary Fig. 1).

**Table 1.** ANOVA results from linear mixed model analysis.

Trait	Term	Sum of squares	Mean squares	NumDF	DenDF	*F*-value	*P*-value	*P* < 0.05
Disease score (0–4)	G	169.5	0.9	189	1,288.6	1.7	5.8044E−07	*
	T	1,707.8	1,707.8	1	1,523.5	3,156.4	0	*
	G × T	142.7	0.8	187	1,518.1	1.4	0.00047086	*
Plant length (cm)	G	8,845.3	46.8	189	1,448.2	1.7	9.2194E−08	*
	T	6,5763.5	65,763.5	1	1,523.9	2,389.3	0	*
	G × T	9,729.5	52.0	187	1,518.2	1.9	1.2054E−10	*
Shoot length (cm)	G	4,975.7	26.3	189	1,327.0	2.2	1.8173E−15	*
	T	29,451.1	29,451.1	1	1,525.0	2,456.3	0	*
	G × T	4,333.1	23.2	187	1,517.7	1.9	2.519E−11	*
Root length (cm)	G	1,109.8	5.9	189	1,493.6	1.1	0.12458869	
	T	7,213.0	7,213.0	1	1,521.6	1,386.3	3.008E−216	*
	G×T	1,665.3	8.9	187	1,516.9	1.7	6.4025E−08	*

G, genotype; T, treatment; G × T, genotype × treatment interaction; NumDF, numerator degrees of freedom; DenDF, denominator degrees of freedom; * significance at *P* < 0.05.

**Table 2.** Summary statistics of traits across treatments.

Trait	Treatment	Min	Mean	SD	Median	Max	$H_{P}^{2}$	$H_{C}^{2}$
Disease score (0–4)	Fg	0.54	3.4	0.44	3.49	4.02	0.3	0.22
	FgCr	0.07	1.42	0.5	1.39	3.24	0.41	0.32
Plant length (cm)	Fg	2.66	9.98	3.3	9.61	24.97	0.45	0.36
	FgCr	10.86	22.26	3.46	22.57	29.29	0.44	0.36
Shoot length (cm)	Fg	0.83	4.79	2.28	4.48	14.07	0.47	0.37
	FgCr	4.07	13.02	2.52	13.05	18.76	0.6	0.51
Root length (cm)	Fg	1.51	5.2	1.23	5.2	10.82	0.36	0.28
	FgCr	4.88	9.27	1.35	9.37	12.27	0.14	0.11

SD, standard deviation; $H_{P}^{2}$, heritability (Piepho and Möhring 2007); $H_{C}^{2}$, heritability (Cullis *et al*. 2006).

### Intertreatment contrasts for *C. rosea* efficacy

Significant (*P* < 0.0001) genotype-by-treatment (G × T) interaction was observed for all the traits, suggesting that the performance of different genotypes varied significantly across the treatments (Table 1). Correlations between treatments showed a weak positive correlation for disease score (*R* = 0.21, *P* = 0.004), a weak negative correlation for root length (*R* = −0.18, *P* = 0.016), and no significant correlation for plant length and shoot length, further highlighting variability in genotype-to-genotype performance across treatments (Fig. 1b, d, f and h).

**Fig. 2.**
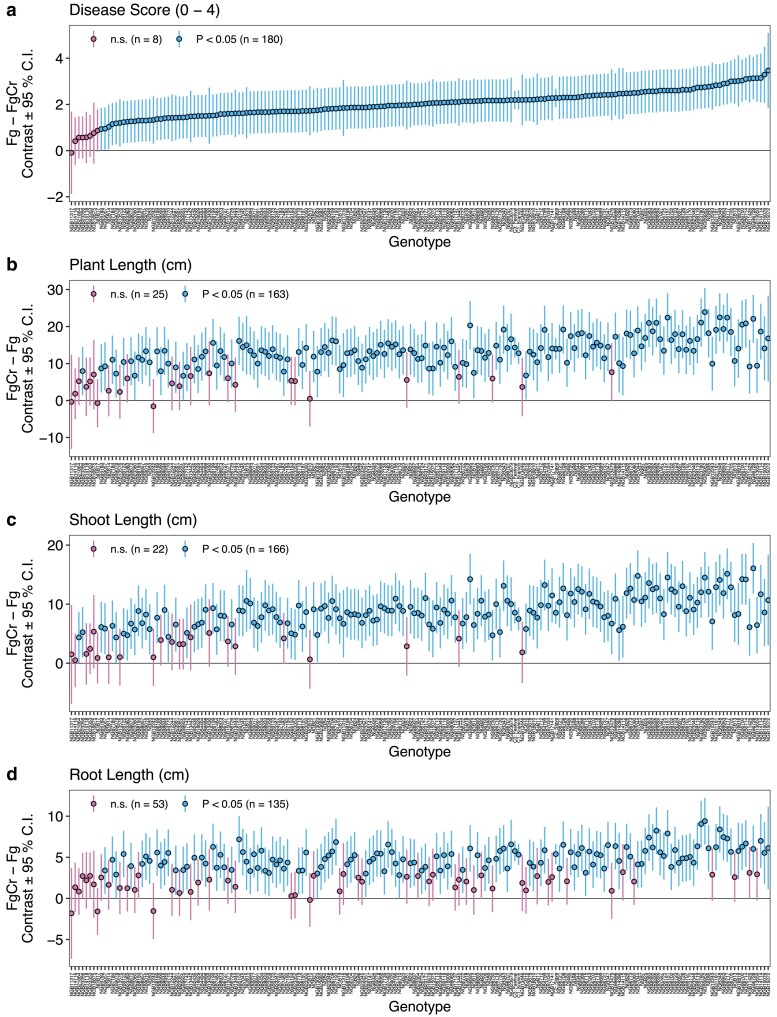
Inter–treatment pairwise contrasts estimates for traits disease score (a), plant length (b), shoot length (c), and root length (d). Inter–treatment pairwise contrasts were estimated for each genotype using post–hoc Tukey tests. Points represent the estimated mean difference between the treatments Fg and FgCr and error bars represent 95% confidence intervals for each genotype. Points with 95% confidence interval overlapping the horizontal line at 0 represent non–significant inter–treatment pairwise contrast.

**Fig. 3.**
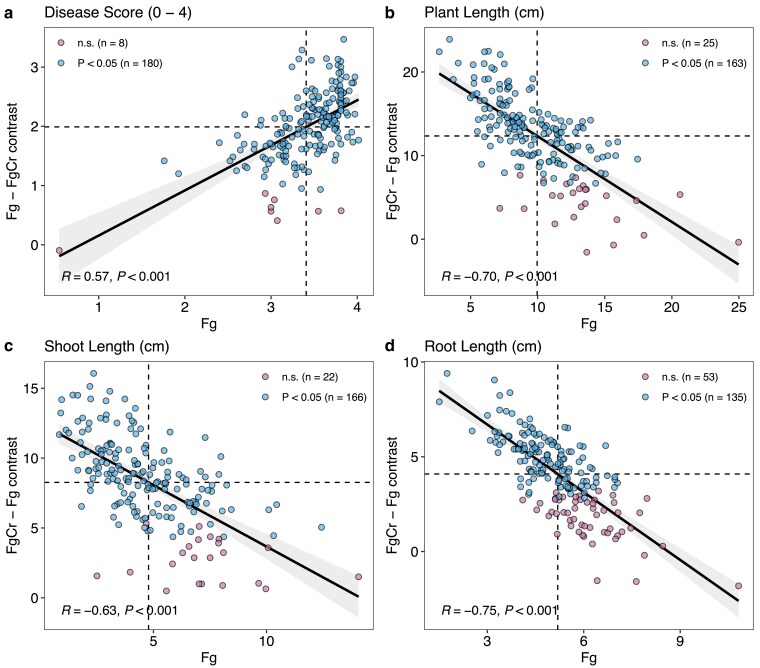
Pearson's correlation between disease score in treatment Fg and *C. rosea* efficacy estimate from inter–treatment pairwise contrast (Fg–FgCr or FgCr–Fg) for traits disease score (a), plant length (b), shoot length (c), and root length (d). Dashed vertical and horizontal lines indicate the mean estimate of the trait in treatment Fg and inter–treatment pairwise contrast (Fg–FgCr or FgCr–Fg) for *C. rosea* efficacy, respectively. Points with pink and blue color represent genotypes with non–significant and significant (*P* < 0.05) inter–treatment pairwise contrast, respectively.

Pairwise contrasts between treatments (Fg–FgCr or FgCr–Fg) for each genotype were used as estimators for *C. rosea* efficacy, i.e. a higher difference in genotype performance between treatments reflects a greater effect of *C. rosea* seed treatment. For disease score, 180 genotypes had a significant (*P* < 0.05) reduction in disease score in the treatment FgCr ranging from 0.93 to 3.47 with an average reduction of 2.05 ± 0.52 (Fig. 2a, Supplementary Table 3). Similarly, most genotypes had a significant (*P* < 0.05) increase in plant length (*n* = 163), shoot length (*n* = 166), and root length (*n* = 135), reflecting the overall treatment effect of *C. rosea* (Fig. 2b–d, Supplementary Table 3). In treatment FgCr, in the presence of *C. rosea*, an average plant length increase of 13.6 ± 3.72 cm (6.68–23.9 cm), an average shoot length increase of 9.00 ± 2.56 cm (4.37–16.1 cm), and an average root length increase of 5.03 ± 1.30 cm (2.83–9.4 cm) was observed. Moreover, the above-described *C. rosea* efficacy estimates from pairwise contrasts were found in significant (*P* < 0.001) correlations with the estimates in the treatment Fg for each trait (Fig. 3). For disease score, a significant moderate positive correlation (*R* = 0.57, *P* < 0.001) was observed between *C. rosea*–mediated biocontrol efficacy to reduce disease and disease susceptibility in the treatment Fg, showing an overall increase in biocontrol efficacy among susceptible genotypes (Fig. 3a). Similarly, negative correlations between treatment Fg estimates and pairwise contrasts for *C. rosea* efficacy for plant length (*R* = −0.7, *P* < 0.001), shoot length (*R* = −0.63, *P* < 0.001), and root length (*R* = −0.75, *P* < 0.001) show that plants with poor growth in treatment Fg had a bigger benefit from *C. rosea* seed treatment (Fig. 3b–d).

### Genome­-wide marker–trait associations

**Fig. 4.**
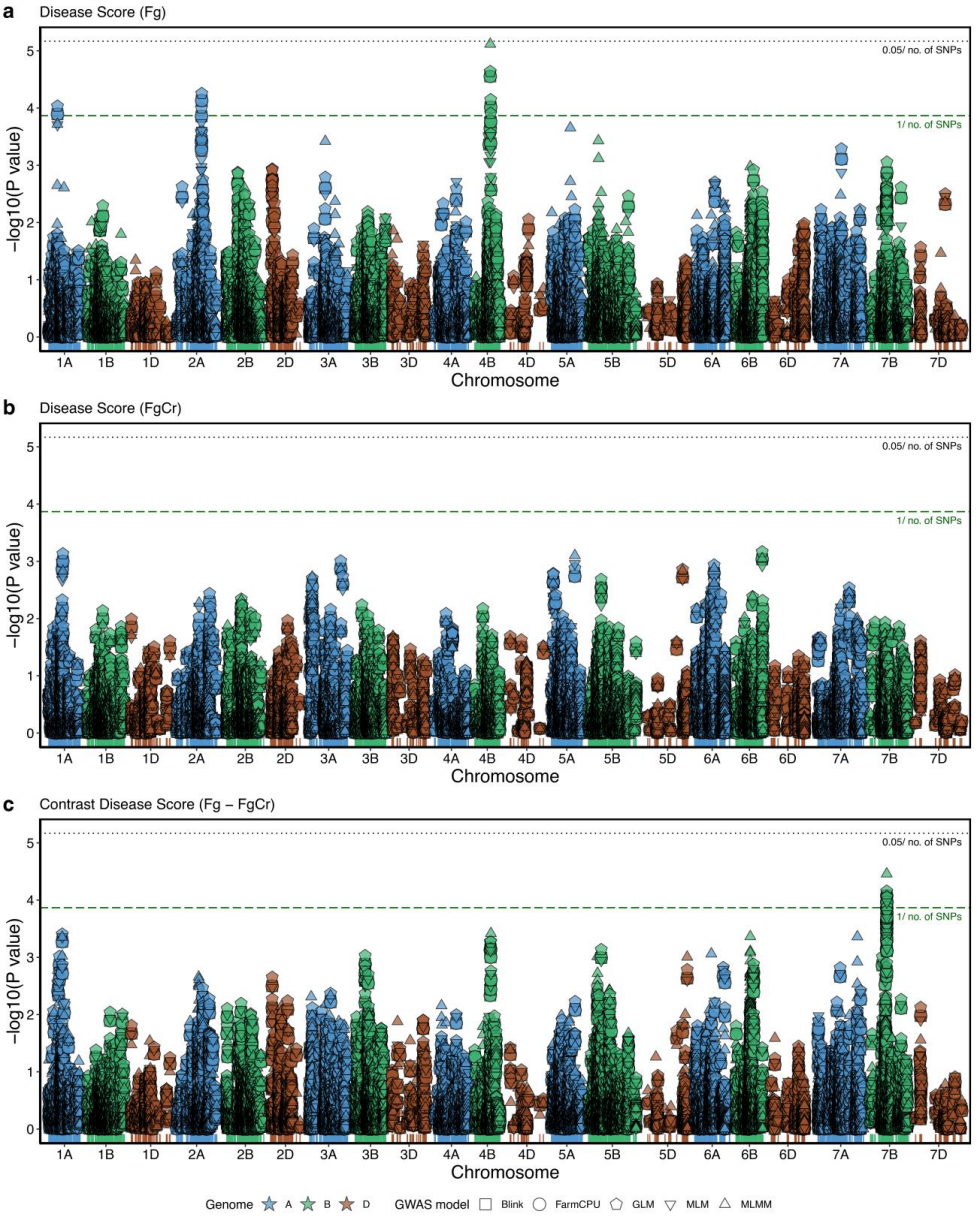
Manhattan plot for marker–trait association for disease score in (a) treatment Fg (*F. graminearum* alone), (b) treatment FgCr (*F. graminearum* on seed treated with *C. rosea*), and (c) disease score contrast (Fg–FgCr) for *C. rosea* efficacy from 5 GWAS models. Dotted line depicts the Bonferroni significance threshold (*P* = 0.00000679, after *P* = 0.05/*n*, where *n* = 7,360 is the number of SNP markers), dashed line depicts negative log threshold (*P* = 0.00014, after *P* = 1/*n*, where *n* = 7,360 is the number of SNP markers).

Phenotypic estimates for genotypes from both treatments, Fg and FgCr, and pairwise contrasts for *C. rosea* efficacy in each trait were assessed for significant (*P* ≤ 0.00014, after *P* ≤ 1/*n*, where *n* = 7,360 is the number of SNP markers retained after filtering) genome-wide marker–trait associations. A total of 181 genotypes for treatment-level associations and 180 genotypes for contrasts had SNP data and phenotypic data and were retained in the analysis. For disease score, significant marker–trait associations were observed in treatment Fg on chromosome 1A at 53 cM, 2A at 115–116 cM, and 4B at 71–73 cM (Fig. 4a). Allele level comparisons at chromosome 1A show no differences in disease scores, significant reduction (*P* < 0.05) in disease scores in genotypes with minor alleles GG and AA for SNP markers BS00089497_51 and Kukri_c40121_373, respectively, at chromosome 2A, and also significant (*P* < 0.05) reduction in disease scores in genotypes with minor alleles TT, GG, and CC for SNP markers BS00096604_51, RFL_Contig2459_2314, and Ku_c33858_325, respectively, at chromosome 4B (Supplementary Fig. 2a–f). No significant SNP marker–trait associations were detected for disease score in treatment FgCr (Fig. 4b), while a significantly associated region was detected for disease score contrast on chromosome 7B at 77–78 cM (Fig. 4c). Allele level comparisons of all 6 associated SNP markers (BobWhite_c3564_81, BS00021972_51, Excalibur_rep_c111629_239, wsnp_Ex_rep_c109138_92064554, BS00010557_51, and wsnp_Ku_rep_c68953_68153061) for disease score contrast showed a significant (*P* < 0.05) increase in *C. rosea* efficacy in genotypes with minor alleles (Supplementary Fig. 2g–l).

For trait plant length, only one significant SNP marker (Ra_c956_2318 on chromosome 7A at 228 cM) was significantly associated with plant length contrast with minor nucleotide T contributing to an increase in plant length due to *C. rosea* seed treatment (Supplementary Figs. 2m and 3). However, the same SNP marker, i.e. Ra_c956_2318 on chromosome 7A at 228 cM, was significantly associated with shoot length contrast but with a nonsignificant effect in allelic comparison (Supplementary Figs. 2o and 4). SNP marker wsnp_Ex_c17914_26681837 on Chr 7D at 139 cM was associated with shoot length in treatment Fg where allele CC was significantly associated with less shoot length (Supplementary Figs. 2n and 4). For root length, one significantly associated region was detected at chromosome 6B at 65 cM in treatment Fg, one significantly associated region at chromosome 7A at 114 cM in treatment FgCr, and no significant association for root length contrast was observed (Supplementary Figs. 2p–r and 5).

### Candidate gene content in SNP-associated genomic regions

Within a stringent interval of ± 100 kb surrounding significant SNP marker–trait associations, localized genes were browsed. Supplementary Table 4 contains all the gene IDs and descriptions for the localized genes. Briefly, for disease score in treatment Fg, 3 genes were found localized with SNP marker BS00089497_51 at chromosome 2A, 2 genes were found localized with SNP marker BS00096604_51 at chromosome 4D, 6 genes were found localized with SNP marker Excalibur_c7026_2635 at chromosome 1A, 3 genes were found localized with SNP marker Ku_c33858_325 at chromosome 4B, 10 genes were found localized with SNP marker Kukri_c40121_373 at chromosome 2A, and 1 gene was found localized with SNP marker RFL_Contig2459_2314 at chromosome 4B. Besides several genes annotated as encoding uncharacterized proteins, 2 genes were predicted to encode kinases, 1 gene was predicted to encode a kinase regulator and 1 gene was predicted to encode an ethylene-responsive transcription factor (Supplementary Table 4).

For disease score contrast (Fg–FgCr), 6 SNP markers were in significant association at chromosome 7B at 77–78 cM. In total, 6 genes were found localized with SNP marker BobWhite_c3564_81, 4 genes were found localized with SNP marker BS00010557_51, 3 genes were found localized with SNP marker BS00021972_51, 1 gene was found localized with SNP marker Excalibur_rep_c111629_239, 4 genes were found localized with SNP marker wsnp_Ex_rep_c109138_92064554, and 3 genes were found localized with SNP marker wsnp_Ku_rep_c68953_68153061. Predicted functions of these gene products included several monooxygenases, transporters, and biosynthesis of secondary metabolites (Supplementary Table 4).

For plant length contrast and shoot length contrast, 9 genes were found localized with SNP marker Ra_c956_2318 at chromosome 7A. These included 2 genes predicted to encode disease resistance proteins, including a Pik-2-like disease resistance protein, and 2 genes predicted to encode receptor kinases (Supplementary Table 4). For shoot length in treatment Fg, 11 genes were found localized with SNP marker wsnp_Ex_c17914_26681837 at chromosome 7D. For root length in treatment Fg, 8 genes were found localized with SNP marker Kukri_c41694_285 at chromosome 6B, and 3 genes were found localized with SNP marker Tdurum_contig15235_951 at chromosome 6B. Moreover, for root length in treatment FgCr, 5 genes were found localized with SNP marker Excalibur_rep_c101407_222 at chromosome 7A (Supplementary Table 4).

## Discussion

In this study, we report genome-wide association analyses of 190 winter wheat genotypes from northern Europe for fusarium foot rot susceptibility and its biocontrol efficacy using *C. rosea*. The same panel of genotypes has previously been explored for genetic variation for resistance to abiotic stress, such as freezing and winter hardiness (Vaitkevičiūtė *et al*. 2023) and drought tolerance (Kumar *et al*. 2020); and to biotic stress, including powdery mildew (Hysing *et al*. 2007; Alemu *et al*. 2021), leaf rust (Hysing *et al*. 2007), yellow rust (Koc *et al*. 2022), fusarium head blight (Zakieh *et al*. 2021), and septoria tritici blotch (Odilbekov *et al*. 2019). Moreover, this panel has been screened for biocontrol efficacy of septoria tritici blotch by *C. rosea* (Chaudhary *et al*. 2024). Here, we show that this panel also serves as a resource for resistance to fusarium foot rot and biocontrol efficacy with *C. rosea*.

We observed significant variation among 190 wheat genotypes for susceptibility to fusarium foot rot caused by *F. graminearum* in the only pathogen treatment. The sand-based bioassay used in this study offers a cost-effective and efficient alternative to field testing for exploring disease severity to fusarium foot rot, as a high correlation (*R* = 0.94, *P* < 0.001) between growth chamber sand bioassay and field conditions were observed for *F. culmorum* disease severity in wheat and barley genotypes (Knudsen *et al*. 1995; Jensen *et al*. 2000). Overall, the genotypes showed a high susceptibility to *F. graminearum* which has been observed in some other works too. Shi *et al*. (2020) observed more than 80% of tested genotypes grouped in susceptible and highly susceptible categories for seedling stage rotting caused by *Fusarium pseudograminearum*. Voss-Fels *et al*. (2018) also observed a high stem discoloration, a metric used to evaluate disease severity caused by *F. graminearum*, in half of 215 tested wheat genotypes. This suggests that the current tested material might not offer full resistance to *F. graminearum* foot rot and might only possess partial resistance with the ability to have reduced symptom development. Kazan and Gardiner (2018) also highlighted the lack of full resistance to fusarium crown rot caused by *F. pseudograminearum*. Disease severity was also found to have a strong negative correlation with other growth-related traits in the study, showing a direct impact on stunting of plant growth and development.

Only a handful of studies have been conducted for *Fusarium* spp.–related ground-level and below-ground diseases in wheat (Li *et al*. 2010; Voss-Fels *et al*. 2018; Liu *et al*. 2021; Malosetti *et al*. 2021). In this study, genome-wide associations revealed significant marker–trait associations for disease score, shoot length, and root length. The SNP marker associations identified at chromosomes 1A, 2A, and 4B for disease score are different from previously identified SNPs in the above-mentioned studies, indicating different genes segregating in the current winter wheat population. Moreover, a significant marker–trait association at chromosome 7D for shoot length and 6B for root length in the presence of pathogen captures segregation at additional locations in the wheat genome. The allelic differences at these markers reveal a significant improvement for growth-related traits and a significant reduction in disease severity, showing the potential for improvement in future breeding programs.

The correlation between resistance to ground-level and below-ground diseases caused by *Fusarium* spp. and resistance to fusarium head blight has been explored previously. Wang *et al*. (2015, 2018) demonstrated a lack of correlation between resistance to fusarium root rot and fusarium head blight and suggested different resistance genes. Similarly, Li *et al*. (2010) observed a very weak correlation (*R* = −0.06–0.27) between fusarium head blight and crown rot severities. Interestingly, Liu *et al*. (2021) observed a significant negative correlation (*R* = −0.263, *P* < 0.01) between fusarium head blight and fusarium seedling blight lesion length. Comparing the results of disease scores from this study to previously conducted FHB using the same panel of winter wheat genotypes (Zakieh *et al*. 2021), we observed no significant correlation (*R* = 0.11, *P* = 0.16, not shown), indicating a different set of resistance genes segregating for *Fusarium* spp.–related disease at seedling stage and flowering stage. This is also further highlighted at the genome-wide level with different regions segregating for disease severity for fusarium foot rot and fusarium head blight between the 2 studies. We note that Zakieh *et al*. (2021) used a mix of 6 *F. graminearum* and 3 *F. culmorum* strains for head infection, while we employed a single *F. graminearum* strain in the current study, which may account for some of the variation. However, as suggested before (Li *et al*. 2010; Liu *et al*. 2021), it is important to have separate screening programs to select for resistance to various *Fusarium* spp. diseases.

One of the main aims of this study was to explore the genetic variation in winter wheat genotypes for the biocontrol efficacy of *C. rosea* in controlling fusarium foot rot. Several previous studies have demonstrated plant-genotype-specific modulation of biocontrol efficacy in various BCA–pathogen interactions, although these studies typically involved limited number of plant genotypes (Smith *et al*. 1999; Ryan *et al*. 2004; Meyer *et al*. 2010; Tucci *et al*. 2011; Rebeka *et al*. 2013; Moraga-Suazo *et al*. 2016; Arkhipov *et al*. 2023). This report, alongside our previous work studying plant genotype effects for biocontrol efficacy of *C. rosea* against septoria tritici blotch (Chaudhary *et al*. 2024), is the exploration of the largest number of plant genotypes for these 3-way interactions among plant, pathogen, and BCA. We observed significant variation among plant genotypes for the biocontrol efficacy of *C. rosea* to control fusarium foot rot. *Clonostachys rosea* is very successful in controlling *Fusarium* spp. diseases at various plant stages in wheat (Knudsen *et al*. 1995; Jensen *et al*. 2000; Roberti *et al*. 2008; Xue *et al*. 2009; Gimeno *et al*. 2021; Abaya *et al*. 2023). However, by identifying the genetic basis in plants for interactions with beneficial microorganisms, the efficacy to reduce the disease can be further enhanced. Due to the large-scale screening of plant genotypes, it was possible to explore the genomic-level segregation among wheat genotypes for biocontrol efficacy. We identified a region at chromosome 7B which is significantly associated with segregation for *C. rosea* biocontrol efficacy and another region on chromosome 7A segregating with *C. rosea* efficacy for shoot length and plant length, suggesting different underlying mechanisms for these traits. Interestingly, association mapping of *C. rosea*–mediated biocontrol efficacy of septoria leaf blotch disease in the same winter wheat collection identified 2 distinct segregating regions on chromosomes 1D and 6B (Chaudhary *et al*. 2024). This shows that plant genotype-mediated biocontrol efficacy can be specific to different pathogens (*F. graminearum* or *Z. tritici*) and/or different plant organs (head or roots).

No overlapping *Fusarium* disease trait associations on chromosome 7B at 77–78 cM are reported in the literature. However, 2 studies using linkage maps reported FHB-related QTLs upstream at 53–66 cM (Eckard *et al*. 2015) and downstream at 92 cM (Wang *et al*. 2023) of the region identified in this study. It should be noted that linkage maps are population-specific, and thus, it is uncertain whether these QTLs are localized within the genomic region identified in this study. The genomic region associated with *C. rosea* biocontrol efficacy on chromosome 7B contained genes predicted to encode various monooxygenases, transporters, and biosynthesis of secondary metabolites. Specifically, a detoxification protein, Detoxification 16-like, belonging to the multidrug and toxic compound extrusion (MATE) transporter family was located in the region. The MATE family is a large multigene family in plants, where the proteins are involved in detoxification of toxic compounds, heavy metals, and disease resistance (Sun *et al*. 2011; Takanashi *et al*. 2014; Watanabe *et al*. 2022). Moreover, 3 different cytochrome P450 (CYPs) encoding genes were located in this region. CYP75B4-like is putatively involved in flavonoid biosynthesis, whereas CYP19-4-like and CYP28 encode for cyclophilin which are involved in protein folding, cell signaling, and also plays a role in immunosuppression in vertebrates and yeast (He *et al*. 2004; Wang and Heitman 2005).

The region on chromosome 7A contained a gene predicted to encode a Pik-2-like disease resistance protein. Pik-2-like disease resistance proteins belong to a known R protein type demonstrated to induce a hypersensitive response in plants to restrict pathogen growth (Ashikawa *et al*. 2008). Interestingly, 2 different Pik-2-like disease resistance protein paralogs are present in a genomic region on chromosome 1D in the same wheat collection, segregating with *C. rosea*–mediated biocontrol efficacy of septoria leaf blotch (Chaudhary *et al*. 2024). The presence of Pik-2-like disease resistance protein genes in different regions segregating with biocontrol efficacy may suggest the ability of wheat genotypes to recognize microbe-associated molecular patterns or microbial effectors and subsequently induce pattern-triggered immunity or effector-triggered immunity to partially contribute to the BCA compatibility trait (Jones and Dangl 2006; Köhl *et al*. 2019; Jensen *et al*. 2021).

It must be emphasized that plant disease resistance must act as the first line of defense in an integrated disease management approach. Therefore, any further manipulation in cultivar development, such as BCA compatibility breeding, should not come at the cost of undermining disease resistance. In our study, we observed a significant positive correlation between disease susceptibility and plant genotype-dependent *C. rosea* biocontrol efficacy, highlighting the better performance of *C. rosea* as a BCA in more susceptible genotypes. Smith *et al*. (1999) also observed a similar trend where better disease suppression by the BCA *B. cereus* was found in less resistant tomato genotypes toward *P. torulosum*. The positive relationship observed between increased disease susceptibility and improved biocontrol efficacy can be attributed to the greater opportunity for disease reduction when higher pathogen loads are present. The correlation is also rather moderate and, therefore, it is possible to select genotypes with lower susceptibility and higher biocontrol efficacy from the population. Moreover, techniques such as GWAS can help in dissecting the traits and break negative linkages, if any, and aid in more precise selection of traits for cultivar improvement. We identified independent associations for disease resistance and *C. rosea* biocontrol efficacy, highlighting the potential for simultaneous breeding for resistance to fusarium foot rot and biocontrol efficacy of *C. rosea* in managing the disease.

## Supplementary Material

jkae240_Supplementary_Data

## Data Availability

Phenotypic and genotypic raw data are available at figshare: https://doi.org/10.25387/g3.26064079. The authors affirm that all data necessary for confirming the conclusions of the article are present within the article, figures, and tables. Supplemental material available at G3 online.

## References

[jkae240-B1] Abaya A, Xue A, Hsiang T. 2023. Systemically induced resistance against disease of wheat caused by *Fusarium graminearum*. Can J Plant Pathol. 45(3):320–329. doi:10.1080/07060661.2023.2177749.

[jkae240-B2] Alemu A, Brazauskas G, Gaikpa DS, Henriksson T, Islamov B, Jørgensen LN, Koppel M, Koppel R, Liatukas Ž, Svensson JT, et al 2021. Genome-wide association analysis and genomic prediction for adult-plant resistance to septoria tritici blotch and powdery mildew in winter wheat. Front Genet. 12:661742. doi:10.3389/fgene.2021.661742. 34054924 PMC8149967

[jkae240-B3] Altschul SF, Gish W, Miller W, Myers EW, Lipman DJ. 1990. Basic local alignment search tool. J Mol Biol. 215(3):403–410. doi:10.1016/S0022-2836(05)80360-2. 2231712

[jkae240-B4] Arkhipov A, Carvalhais LC, Schenk PM. 2023. PGPR control *Phytophthora capsici* in tomato through induced systemic resistance, early hypersensitive response and direct antagonism in a cultivar-specific manner. Eur J Plant Pathol. 167(4):811–832. doi:10.1007/s10658-023-02734-8.

[jkae240-B5] Ashikawa I, Hayashi N, Yamane H, Kanamori H, Wu J, Matsumoto T, Ono K, Yano M. 2008. Two adjacent nucleotide-binding site-leucine-rich repeat class genes are required to confer Pikm-specific rice blast resistance. Genetics. 180(4):2267–2276. doi:10.1534/genetics.108.095034. 18940787 PMC2600957

[jkae240-B6] Barnett HL, Lilly VG. 1962. A destructive mycoparasite, *Gliocladium roseum*. Mycologia. 54(1):72–77. doi:10.1080/00275514.1962.12024980.

[jkae240-B7] Barzman M, Bàrberi P, Birch ANE, Boonekamp P, Dachbrodt-Saaydeh S, Graf B, Hommel B, Jensen JE, Kiss J, Kudsk P, et al 2015. Eight principles of integrated pest management. Agron Sustain Dev. 35(4):1199–1215. doi:10.1007/s13593-015-0327-9.

[jkae240-B8] Bates D, Mächler M, Bolker B, Walker S. 2015. Fitting linear mixed-effects models using {lme4}. J Stat Softw. 67(1):1–48. doi:10.18637/jss.v067.i01.

[jkae240-B9] Becher R, Miedaner T, Wirsel SGR. 2013. 8 Biology, diversity, and management of FHB-causing *Fusarium* species in small-grain cereals. In: Kempken F, editors. Agricultural Applications. Berlin: Springer. p. 199–241. 10.1007/978-3-642-36821-9_8.

[jkae240-B10] Benjamini Y, Hochberg Y. 1995. Controlling the false discovery rate: a practical and powerful approach to multiple testing. J R Stat Soc Ser B Methodol. 57(1):289–300. doi:10.1111/j.2517-6161.1995.tb02031.x.

[jkae240-B11] Buerstmayr M, Steiner B, Buerstmayr H. 2020. Breeding for Fusarium head blight resistance in wheat—progress and challenges. Plant Breed. 139(3):429–454. doi:10.1111/pbr.12797.

[jkae240-B12] Chaudhary S, Zakieh M, Dubey M, Jensen DF, Grenville-Briggs L, Chawade A, Karlsson M. 2024. Plant genotype-specific modulation of Clonostachys rosea-mediated biocontrol of septoria tritici blotch disease on wheat. bioRxiv 029983. https://doi:10.1101/2024.05.28.596162 , preprint: not peer reviewed. 10.1186/s12870-025-06620-9 PMC12049020 40316900

[jkae240-B13] Collinge DB, Jensen DF, Rabiey M, Sarrocco S, Shaw MW, Shaw RH. 2022. Biological control of plant diseases – what has been achieved and what is the direction? Plant Pathol. 71(5):1024–1047. doi:10.1111/ppa.13555.

[jkae240-B14] Cullis BR, Smith AB, Coombes NE. 2006. On the design of early generation variety trials with correlated data. J Agric Biol Environ Stat. 11(4):381–393. doi:10.1198/108571106X154443/METRICS.

[jkae240-B15] Dean R, Van Kan JAL, Pretorius ZA, Hammond-Kosack KE, Di Pietro A, Spanu PD, Rudd JJ, Dickman M, Kahmann R, Ellis J, et al 2012. The top 10 fungal pathogens in molecular plant pathology. Mol Plant Pathol. 13(4):414–430. doi:10.1111/j.1364-3703.2011.00783.x. 22471698 PMC6638784

[jkae240-B16] Eckard JT, Glover KD, Mergoum M, Anderson JA, Gonzalez-Hernandez JL. 2015. Multiple Fusarium head blight resistance loci mapped and pyramided onto elite spring wheat Fhb1 backgrounds using an IBD-based linkage approach. Euphytica. 204(1):63–79. doi:10.1007/s10681-014-1333-8.

[jkae240-B17] European Commission . 2022. Proposal for a regulation of the European Parliament and of the Council on the sustainable use of plant protection products and amending Regulation (EU) 2021/2115. https://eur-lex.europa.eu/legal-content/EN/TXT/?uri=CELEX%3A52022PC0305.

[jkae240-B18] European Union . 2009. Directive 2009/128/EC of the European Parliament and of the Council of 21 October 2009 establishing a framework for Community action to achieve the sustainable use of pesticides. https://eur-lex.europa.eu/eli/dir/2009/128/oj.

[jkae240-B19] FAO . 2022. Pesticides Use, Pesticides Trade and Pesticides Indicators: Global, Regional and Country Trends, 1990–2020. Rome (Italy): FAO (FAOSTAT analytical briefs).

[jkae240-B20] Fortune Business Insights . 2022. Biopesticides market size, share & COVID-19 impact analysis, by type (bioinsecticide, biofungicide, bionematicide, and others), by source (microbials and biochemicals), by mode of application (foliar application, seed treatment, soil treatment, and others), by crop (cereals, oilseeds, fruits & vegetables, and others), and regional forecast, 2022–2029. https://www.fortunebusinessinsights.com/industry-reports/biopesticides-market-100073.

[jkae240-B21] Gimeno A, Stanley CE, Ngamenie Z, Hsung MH, Walder F, Schmieder SS, Bindschedler S, Junier P, Keller B, Vogelgsang S. 2021. A versatile microfluidic platform measures hyphal interactions between *Fusarium graminearum* and *Clonostachys rosea* in real-time. Commun Biol. 4(1):262. doi:10.1038/s42003-021-01767-1. 33637874 PMC7910300

[jkae240-B22] Gould F, Brown ZS, Kuzma J. 2018. Wicked evolution: can we address the sociobiological dilemma of pesticide resistance? Science. 360(6390):728–732. doi:10.1126/science.aar3780. 29773742

[jkae240-B23] Graves S, Piepho H-P, Selzer L, et al multcompView: Visualizations of Paired Comparisons. doi:10.32614/CRAN.package.multcompView. 2023.

[jkae240-B24] Han P, Zhang X, Xu D, Zhang B, Lai D, Zhou L. 2020. Metabolites from *Clonostachys* fungi and their biological activities. J Fungi. 6(4):229. doi:10.3390/jof6040229. PMC7712584 33081356

[jkae240-B25] He Z, Li L, Luan S. 2004. Immunophilins and parvulins. superfamily of peptidyl prolyl isomerases in Arabidopsis. Plant Physiol. 134(4):1248–1267. doi:10.1104/pp.103.031005. 15047905 PMC419802

[jkae240-B26] Hothorn T, Bretz F, Westfall P. 2008. Simultaneous inference in general parametric models. Biom J. 50(3):346–363. doi:10.1002/bimj.200810425. 18481363

[jkae240-B27] Huang M, Liu X, Zhou Y, Summers RM, Zhang Z. 2019. BLINK: a package for the next level of genome-wide association studies with both individuals and markers in the millions. GigaScience. 8(2):giy151. doi:10.1093/GIGASCIENCE/GIY154. PMC6365300 30535326

[jkae240-B28] Hysing S-C, Merker A, Liljeroth E, Koebner RMD, Zeller FJ, Hsam SLK. 2007. Powdery mildew resistance in 155 Nordic bread wheat cultivars and landraces. Hereditas. 144(3):102–119. doi:10.1111/j.2007.0018-0661.01991.x. 17663702

[jkae240-B31] Jensen B, Knudsen IMB, Jensen DF. 2000. Biological seed treatment of cereals with fresh and long-term stored formulations of *Clonostachys rosea*: biocontrol efficacy against *Fusarium culmorum*. Eur J Plant Pathol. 106(3):233–242. doi:10.1023/A:1008794626600.

[jkae240-B29] Jensen DF, Dubey M, Jensen B, Karlsson M. 2021. *Clonostachys rosea* to control plant diseases. In: Köhl J, Ravensberg WJ, editors. Microbial bioprotectants for plant disease management. Burleigh Dodds Science Publishing Limited. p. 429–472. doi: 10.19103/AS.2021.0093.14.

[jkae240-B30] Jensen DF, Karlsson M, Lindahl BD. 2017. Chapter 38 Fungal–fungal interactions: from natural ecosystems to managed plant production, with emphasis on biological control of plant diseases. In: Dighton J, White J, editors. The fungal community: its organization and role in the ecosystem. 4th ed. Boca Raton: CRC Press. p. 549–562. 10.1201/9781315119496-39.

[jkae240-B32] Jones JDG, Dangl JL. 2006. The plant immune system. Nature. 444(7117):323–329. doi:10.1038/nature05286. 17108957

[jkae240-B33] Kamou NN, Cazorla F, Kandylas G, Lagopodi AL. 2020. Induction of defense-related genes in tomato plants after treatments with the biocontrol agents *Pseudomonas chlororaphis* ToZa7 and *Clonostachys rosea* IK726. Arch Microbiol. 202(2):257–267. doi:10.1007/s00203-019-01739-4. 31605156

[jkae240-B34] Karlsson M, Durling MB, Choi J, Kosawang C, Lackner G, Tzelepis GD, Nygren K, Dubey MK, Kamou N, Levasseur A, et al 2015. Insights on the evolution of mycoparasitism from the genome of *Clonostachys rosea*. Genome Biol Evol. 7(2):465–480. doi:10.1093/gbe/evu292. 25575496 PMC4350171

[jkae240-B35] Karlsson I, Persson P, Friberg H. 2021. Fusarium head blight from a microbiome perspective. Front Microbiol. 12:628373. doi:10.3389/fmicb.2021.628373. 33732223 PMC7956947

[jkae240-B36] Karlsson Green K, Stenberg JA, Lankinen Å. 2020. Making sense of integrated pest management (IPM) in the light of evolution. Evol Appl. 13(8):1791–1805. doi:10.1111/eva.13067. 32908586 PMC7463341

[jkae240-B37] Kazan K, Gardiner DM. 2018. Fusarium crown rot caused by *Fusarium pseudograminearum* in cereal crops: recent progress and future prospects. Mol Plant Pathol. 19(7):1547–1562. doi:10.1111/mpp.12639. 29105256 PMC6638152

[jkae240-B38] Kenward MG, Roger JH. 1997. Small sample inference for fixed effects from restricted maximum likelihood. Biometrics. 53(3):983. doi:10.2307/2533558. 9333350

[jkae240-B39] Knudsen IMB, Hockenhull J, Jensen DF. 1995. Biocontrol of seedling diseases of barley and wheat caused by *Fusarium culmorum* and *Bipolaris sorokiniana*: effects of selected fungal antagonists on growth and yield components. Plant Pathol. 44(3):467–477. doi:10.1111/j.1365-3059.1995.tb01669.x.

[jkae240-B40] Koc A, Odilbekov F, Alamrani M, Henriksson T, Chawade A. 2022. Predicting yellow rust in wheat breeding trials by proximal phenotyping and machine learning. Plant Methods. 18(1):30. doi:10.1186/s13007-022-00868-0. 35292072 PMC8922805

[jkae240-B41] Köhl J, Kolnaar R, Ravensberg WJ. 2019. Mode of action of microbial biological control agents against plant diseases: relevance beyond efficacy. Front Plant Sci. 10:845. doi:10.3389/fpls.2019.00845. 31379891 PMC6658832

[jkae240-B42] Kumar D, Kushwaha S, Delvento C, Liatukas Ž, Vivekanand V, Svensson JT, Henriksson T, Brazauskas G, Chawade A. 2020. Affordable phenotyping of winter wheat under field and controlled conditions for drought tolerance. Agronomy. 10(6):882. doi:10.3390/agronomy10060882.

[jkae240-B43] Kuznetsova A, Brockhoff PB, Christensen RHB. 2017. lmerTest package: tests in linear mixed effects models. J Stat Softw. 82(13):1–26. doi:10.18637/jss.v082.i13.

[jkae240-B44] Laidig F, Feike T, Klocke B, Macholdt J, Miedaner T, Rentel D, Piepho HP. 2021. Long-term breeding progress of yield, yield-related, and disease resistance traits in five cereal crops of German variety trials. Theor Appl Genet. 134(12):3805–3827. doi:10.1007/s00122-021-03929-5. 34652455 PMC8580907

[jkae240-B45] Leišová-Svobodová L, Chrpová J, Hermuth J, Dotlačil L. 2020. Quo vadis wheat breeding: a case study in Central Europe. Euphytica. 216(9):141. doi:10.1007/s10681-020-02670-2.

[jkae240-B46] Lenth RV. 2023. emmeans: Estimated Marginal Means, aka Least-Squares Means. doi:10.32614/CRAN.package.emmeans.

[jkae240-B47] Li HB, Xie GQ, Ma J, Liu GR, Wen SM, Ban T, Chakraborty S, Liu CJ. 2010. Genetic relationships between resistances to Fusarium head blight and crown rot in bread wheat (*Triticum aestivum* L.). Theor Appl Genet. 121(5):941–950. doi:10.1007/s00122-010-1363-0. 20535443

[jkae240-B48] Liu X, Huang M, Fan B, Buckler ES, Zhang Z. 2016. Iterative usage of fixed and random effect models for powerful and efficient genome-wide association studies. PLoS Genet. 12(2):e1005767. doi:10.1371/journal.pgen.1005767. 26828793 PMC4734661

[jkae240-B49] Liu Y, Zhu G, Zhu Z, Chen L, Niu H, He W, Tong H, Song J, Zhang Y, Ma D, et al 2021. Investigation and genome-wide association analysis of fusarium seedling blight resistance in Chinese elite wheat lines. Front Plant Sci. 12:777494. doi:10.3389/fpls.2021.777494. 34868179 PMC8635748

[jkae240-B50] Malosetti M, Zwep LB, Forrest K, van Eeuwijk FA, Dieters M. 2021. Lessons from a GWAS study of a wheat pre-breeding program: pyramiding resistance alleles to Fusarium crown rot. Theor Appl Genet. 134(3):897–908. doi:10.1007/s00122-020-03740-8. 33367942 PMC7925461

[jkae240-B51] Meyer JB, Lutz MP, Frapolli M, Péchy-Tarr M, Rochat L, Keel C, Défago G, Maurhofer M. 2010. Interplay between wheat cultivars, biocontrol pseudomonads, and soil. Appl Environ Microbiol. 76(18):6196–6204. doi:10.1128/AEM.00752-10. 20675454 PMC2937482

[jkae240-B52] Moraga-Suazo P, Sanfuentes E, Le-Feuvre R. 2016. Induced systemic resistance triggered by *Clonostachys rosea* against *Fusarium circinatum* in *Pinus radiata*. For Res Open Access. 5(2):1000174. doi:10.4172/2168-9776.1000174.

[jkae240-B53] Odilbekov F, Armoniené R, Koc A, Svensson J, Chawade A. 2019. GWAS-assisted genomic prediction to predict resistance to septoria tritici blotch in Nordic winter wheat at seedling stage. Front Genet. 10:1224. doi:10.3389/fgene.2019.01224. 31850073 PMC6901976

[jkae240-B54] Piepho H-P, Möhring J. 2007. Computing heritability and selection response from unbalanced plant breeding trials. Genetics. 177(3):1881–1888. doi:10.1534/genetics.107.074229. 18039886 PMC2147938

[jkae240-B55] Prashar P, Vandenberg A. 2017. Genotype-specific responses to the effects of commercial *Trichoderma* formulations in lentil (*Lens culinaris* ssp. *culinaris*) in the presence and absence of the oomycete pathogen *Aphanomyces euteiches*. Biocontrol Sci Technol. 27(10):1123–1144. doi:10.1080/09583157.2017.1376035.

[jkae240-B56] Price AL, Patterson NJ, Plenge RM, Weinblatt ME, Shadick NA, Reich D. 2006. Principal components analysis corrects for stratification in genome-wide association studies. Nat Genet. 38(8):904–909. doi:10.1038/ng1847. 16862161

[jkae240-B57] R Core Team . 2023. R: A Language and Environment for Statistical Computing. Vienna, Austria: R Foundation for Statistical Computing.

[jkae240-B58] Rebeka G, Shimelis H, Laing MD, Tongoona P, Mandefro N. 2013. Evaluation of Sorghum genotypes compatibility with *Fusarium oxysporum* under *Striga* infestation. Crop Sci. 53(2):385–393. doi:10.2135/cropsci2012.02.0101.

[jkae240-B59] Roberti R, Veronesi A, Cesari A, Cascone A, Di Berardino I, Bertini L, Caruso C. 2008. Induction of PR proteins and resistance by the biocontrol agent *Clonostachys rosea* in wheat plants infected with *Fusarium culmorum*. Plant Sci. 175(3):339–347. doi:10.1016/j.plantsci.2008.05.003.

[jkae240-B60] Ryan AD, Kinkel LL, Schottel JL. 2004. Effect of pathogen isolate, potato cultivar, and antagonist strain on potato scab severity and biological control. Biocontrol Sci Technol. 14(3):301–311. doi:10.1080/09583150410001665187.

[jkae240-B61] Saraiva RM, Borges ÁV, Borel FC, Maffia LA. 2020. Compounds produced by *Clonostachys rosea* deleterious to *Botrytis cinerea*. Braz J Agric. 95(1):34. doi:10.37856/bja.v95i1.3711.

[jkae240-B62] Schmidt J, Dotson BR, Schmiderer L, van Tour A, Kumar B, Marttila S, Fredlund KM, Widell S, Rasmusson AG. 2020. Substrate and plant genotype strongly influence the growth and gene expression response to *Trichoderma afroharzianum* T22 in sugar beet. Plants. 9(8):1005. doi:10.3390/plants9081005. 32784636 PMC7464433

[jkae240-B63] Schroers H-J, Samuels GJ, Seifert KA, Gams W. 1999. Classification of the mycoparasite *Gliocladium roseum* in *Clonostachys* as *C. rosea*, its relationship to *Bionectria ochroleuca*, and notes on other *Gliocladium*-like fungi. Mycologia. 91(2):365–385. doi:10.1080/00275514.1999.12061028.

[jkae240-B64] Segura V, Vilhjálmsson BJ, Platt A, Korte A, Seren Ü, Long Q, Nordborg M. 2012. An efficient multi-locus mixed-model approach for genome-wide association studies in structured populations. Nat Genet. 44(7):825–830. doi:10.1038/ng.2314. 22706313 PMC3386481

[jkae240-B65] Shi S, Zhao J, Pu L, Sun D, Han D, Li C, Feng X, Fan D, Hu X. 2020. Identification of new sources of resistance to crown rot and fusarium head blight in wheat. Plant Dis. 104(7):1979–1985. doi:10.1094/PDIS-10-19-2254-RE. 32384253

[jkae240-B66] Smith KP, Goodman RM. 1999. Host variation for interactions with beneficial plant-associated microbes. Annu Rev Phytopathol. 37(1):473–491. doi:10.1146/annurev.phyto.37.1.473. 11701832

[jkae240-B67] Smith KP, Handelsman J, Goodman RM. 1999. Genetic basis in plants for interactions with disease-suppressive bacteria. Proc Natl Acad Sci U S A. 96(9):4786–4790. doi:10.1073/pnas.96.9.4786. 10220371 PMC21769

[jkae240-B68] Stenberg JA, Heil M, Åhman I, Björkman C. 2015. Optimizing crops for biocontrol of pests and disease. Trends Plant Sci. 20(11):698–712. doi:10.1016/j.tplants.2015.08.007. 26447042

[jkae240-B69] Stenberg JA, Sundh I, Becher PG, Björkman C, Dubey M, Egan PA, Friberg H, Gil JF, Jensen DF, Jonsson M, et al 2021. When is it biological control? A framework of definitions, mechanisms, and classifications. J Pest Sci. 94(3):665–676. doi:10.1007/s10340-021-01354-7.

[jkae240-B70] Strandberg G, Andersson B, Berlin A. 2024. Plant pathogen infection risk and climate change in the Nordic and Baltic countries. Environ Res Commun. 6(3):031008. doi:10.1088/2515-7620/ad352a.

[jkae240-B71] Sun X, Gilroy EM, Chini A, Nurmberg PL, Hein I, Lacomme C, Birch PRJ, Hussain A, Yun B-W, Loake GJ. 2011. ADS1 encodes a MATE-transporter that negatively regulates plant disease resistance. New Phytol. 192(2):471–482. doi:10.1111/j.1469-8137.2011.03820.x. 21762165

[jkae240-B72] Sutton JC, Li D-W, Peng G, Yu H, Zhang P, Valdebenito-Sanhueza RM. 1997. *Gliocladium roseum*: a versatile adversary of *Botrytis cinerea* in crops. Plant Dis. 81(4):316–328. doi:10.1094/PDIS.1997.81.4.316. 30861808

[jkae240-B73] Takanashi K, Shitan N, Yazaki K. 2014. The multidrug and toxic compound extrusion (MATE) family in plants. Plant Biotechnol. 31(5):417–430. doi:10.5511/plantbiotechnology.14.0904a.

[jkae240-B74] Trail F, Common R. 2000. Perithecial development by *Gibberella zeae*: a light microscopy study. Mycologia. 92(1):130–138. doi:10.1080/00275514.2000.12061137.

[jkae240-B75] Tucci M, Ruocco M, De Masi L, De Palma M, Lorito M. 2011. The beneficial effect of *Trichoderma* spp. on tomato is modulated by the plant genotype. Mol Plant Pathol. 12(4):341–354. doi:10.1111/j.1364-3703.2010.00674.x. 21453429 PMC6640367

[jkae240-B76] Tudi M, Daniel Ruan H, Wang L, Lyu J, Sadler R, Connell D, Chu C, Phung DT. 2021. Agriculture development, pesticide application and its impact on the environment. Int J Environ Res Public Health. 18(3):1112. doi:10.3390/ijerph18031112. 33513796 PMC7908628

[jkae240-B77] Vaitkevičiūtė G, Chawade A, Lillemo M, Liatukas Ž, Aleliūnas A, Armonienė R. 2023. Genome-wide association analysis of freezing tolerance and winter hardiness in winter wheat of Nordic origin. Plants. 12(23):4014. doi:10.3390/plants12234014. 38068649 PMC10708462

[jkae240-B78] Voss-Fels KP, Qian L, Gabur I, Obermeier C, Hickey LT, Werner CR, Kontowski S, Frisch M, Friedt W, Snowdon RJ, et al 2018. Genetic insights into underground responses to *Fusarium graminearum* infection in wheat. Sci Rep. 8(1):13153. doi:10.1038/s41598-018-31544-w. 30177750 PMC6120866

[jkae240-B79] Voss-Fels KP, Stahl A, Wittkop B, Lichthardt C, Nagler S, Rose T, Chen T-W, Zetzsche H, Seddig S, Majid Baig M, et al 2019. Breeding improves wheat productivity under contrasting agrochemical input levels. Nat Plants. 5(7):706–714. doi:10.1038/s41477-019-0445-5. 31209285

[jkae240-B86] Wang D, Zhao Y, Zhao X, Ji M, Guo X, Tian J, Chen G, Deng Z. 2023. Genome-wide association analysis of type II resistance to Fusarium head blight in common wheat. PeerJ. 11:e15906. doi:10.7717/peerj.15906. 37750077 PMC10518165

[jkae240-B85] Wang J, Zhang Z. 2021. GAPIT version 3: boosting power and accuracy for genomic association and prediction. Genomics Proteomics Bioinformatics. 19(4):629–640. doi:10.1016/j.gpb.2021.08.005. 34492338 PMC9121400

[jkae240-B84] Wang M, Yan J, Zhao J, Song W, Zhang X, Xiao Y, Zheng Y. 2012. Genome-wide association study (GWAS) of resistance to head smut in maize. Plant Sci. 196:125–131. doi:10.1016/j.plantsci.2012.08.004. 23017907

[jkae240-B81] Wang P, Heitman J. 2005. The cyclophilins. Genome Biol. 6(7):226. doi:10.1186/gb-2005-6-7-226. 15998457 PMC1175980

[jkae240-B80] Wang Q, Chen X, Chai X, Xue D, Zheng W, Shi Y, Wang A. 2019. The involvement of jasmonic acid, ethylene, and salicylic acid in the signaling pathway of *Clonostachys rosea*-induced resistance to gray mold disease in tomato. Phytopathology. 109(7):1102–1114. doi:10.1094/PHYTO-01-19-0025-R. 30880572

[jkae240-B82] Wang Q, Shao B, Shaikh FI, Friedt W, Gottwald S. 2018. Wheat resistances to fusarium root rot and head blight are both associated with deoxynivalenol- and jasmonate-related gene expression. Phytopathology. 108(5):602–616. doi:10.1094/PHYTO-05-17-0172-R. 29256831

[jkae240-B83] Wang Q, Vera Buxa S, Furch A, Friedt W, Gottwald S. 2015. Insights into *Triticum aestivum* seedling root rot caused by *Fusarium graminearum*. Mol Plant-Microbe Interactions. 28(12):1288–1303. doi:10.1094/MPMI-07-15-0144-R. 26325125

[jkae240-B87] Watanabe M, Otagaki S, Matsumoto S, Shiratake K. 2022. Genome-wide analysis of multidrug and toxic compound extruction transporters in grape. Front Plant Sci. 13:892638. doi:10.3389/fpls.2022.892638. 35909729 PMC9330396

[jkae240-B88] Wickham H, Averick M, Bryan J, Chang W, McGowan L, François R, Grolemund G, Hayes A, Henry L, Hester J, et al 2019. Welcome to the tidyverse. J Open Source Softw. 4(43):1686. doi:10.21105/joss.01686.

[jkae240-B89] Xue AG, Voldeng HD, Savard ME, Fedak G, Tian X, Hsiang T. 2009. Biological control of fusarium head blight of wheat with *Clonostachys rosea* strain ACM941. Can J Plant Pathol. 31(2):169–179. doi:10.1080/07060660909507590.

[jkae240-B90] Yang J, Manolio TA, Pasquale LR, Boerwinkle E, Caporaso N, Cunningham JM, De Andrade M, Feenstra B, Feingold E, Hayes MG, et al 2011. Genome partitioning of genetic variation for complex traits using common SNPs. Nat Genet. 43(6):519–525. doi:10.1038/ng.823. 21552263 PMC4295936

[jkae240-B91] Yu J, Pressoir G, Briggs WH, Bi IV, Yamasaki M, Doebley JF, McMullen MD, Gaut BS, Nielsen DM, Holland JB, et al 2006. A unified mixed-model method for association mapping that accounts for multiple levels of relatedness. Nat Genet. 38(2):203–208. doi:10.1038/ng1702. 16380716

[jkae240-B92] Zakieh M, Gaikpa DS, Leiva Sandoval F, Alamrani M, Henriksson T, Odilbekov F, Chawade A. 2021. Characterizing winter wheat germplasm for fusarium head blight resistance under accelerated growth conditions. Front Plant Sci. 12:705006. doi:10.3389/fpls.2021.705006. 34512690 PMC8425451

[jkae240-B93] Zetzsche H, Friedt W, Ordon F. 2020. Breeding progress for pathogen resistance is a second major driver for yield increase in German winter wheat at contrasting N levels. Sci Rep. 10(1):20374. doi:10.1038/s41598-020-77200-0. 33230232 PMC7683597

