# Supplementary figures and images for: Genotypic variation in winter wheat for fusarium foot rot and its biocontrol using Clonostachys rosea
Source: G3 (Bethesda). 2024 Oct 7;14(12):jkae240. doi: 10.1093/g3journal/jkae240 (PMC11631536; doi:10.1093/g3journal/jkae240)

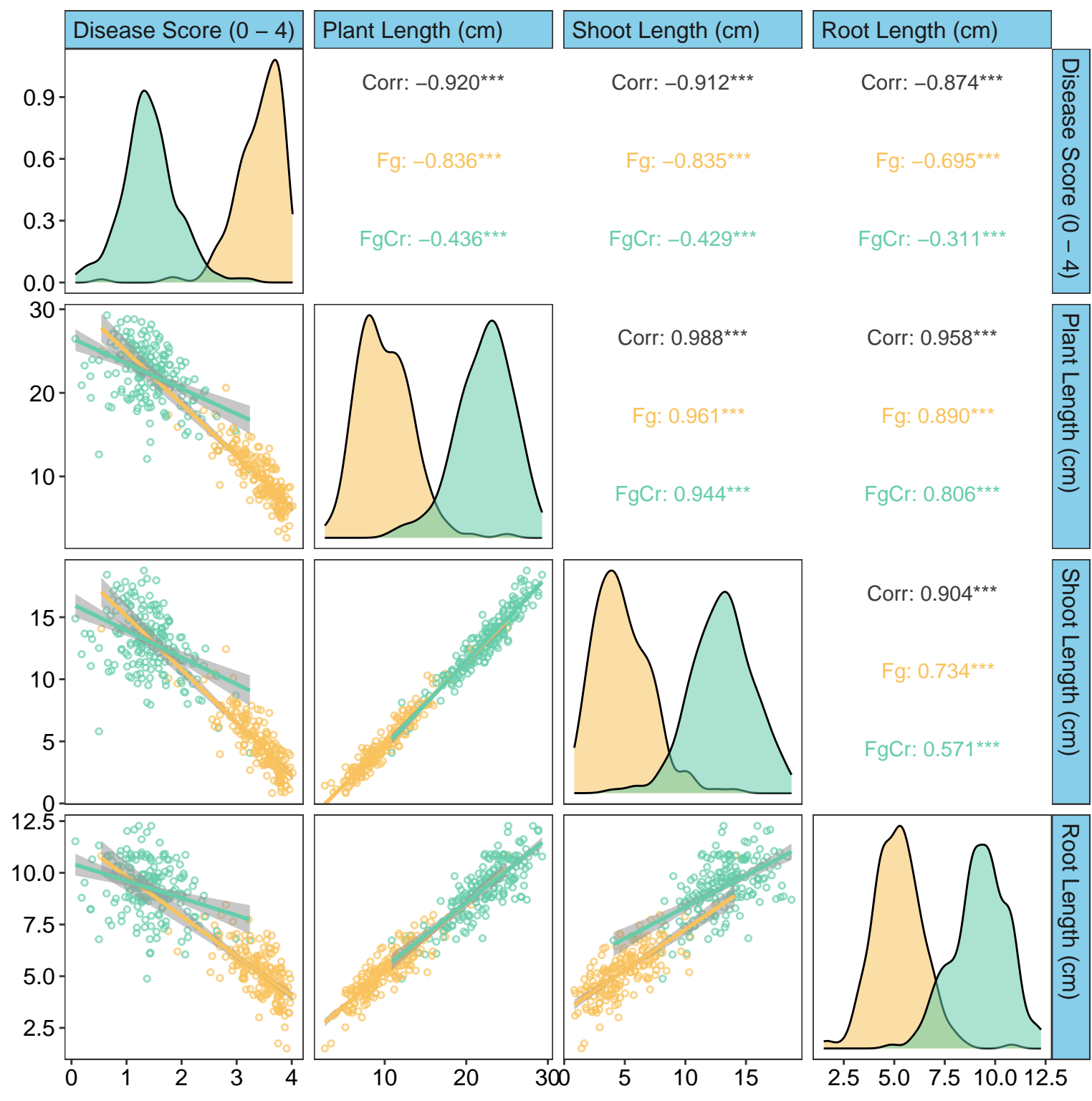

Supplement: jkae240_Supplementary_Data [file jkae240_supplementary_data.zip › Figure_S1_G3-2024-405205.pdf]

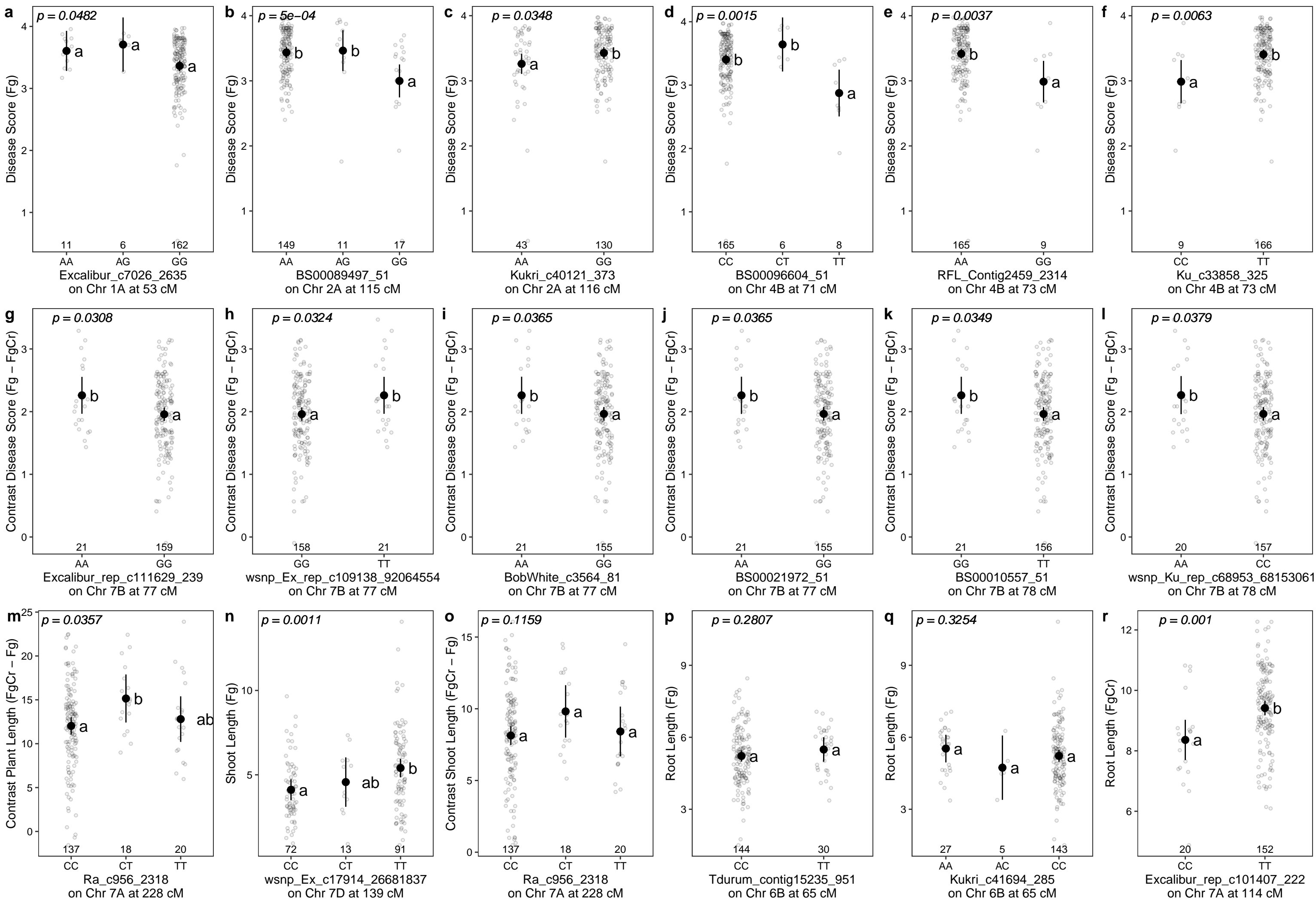

Supplement: jkae240_Supplementary_Data [file jkae240_supplementary_data.zip › Figure_S2_G3-2024-405205.pdf]

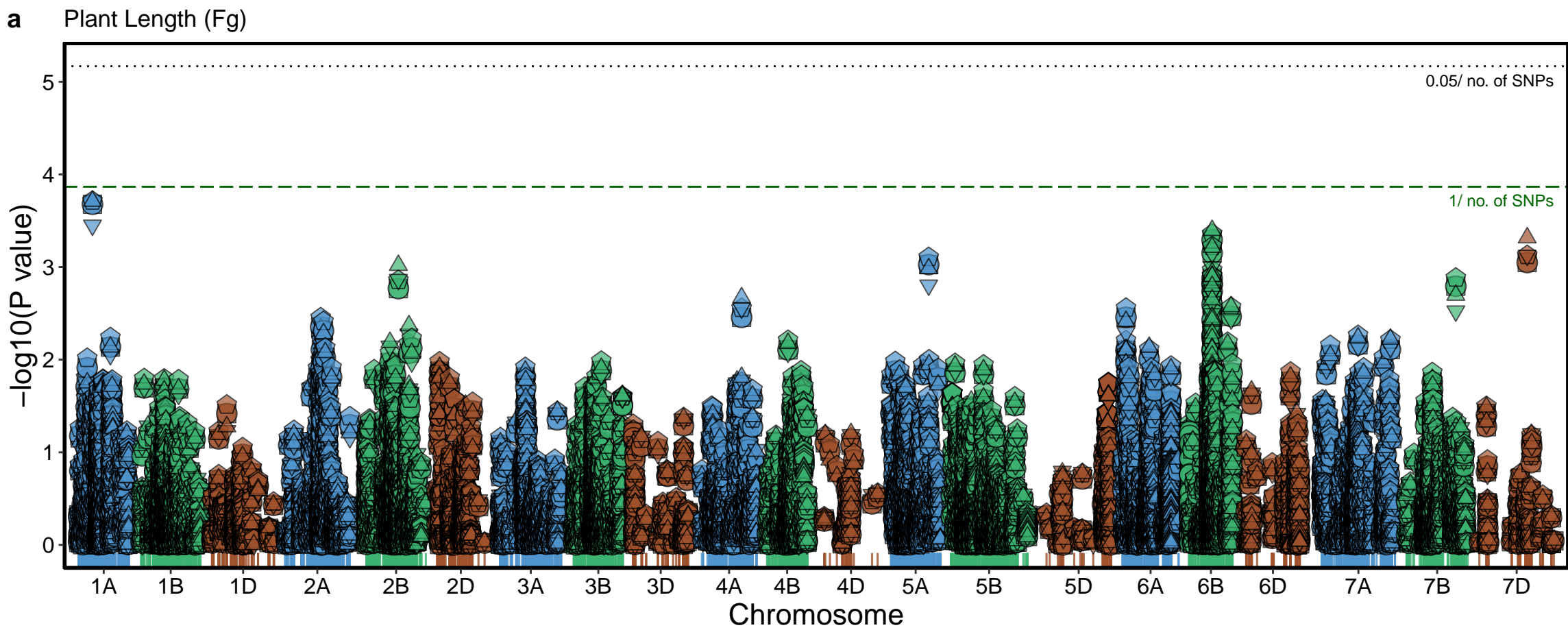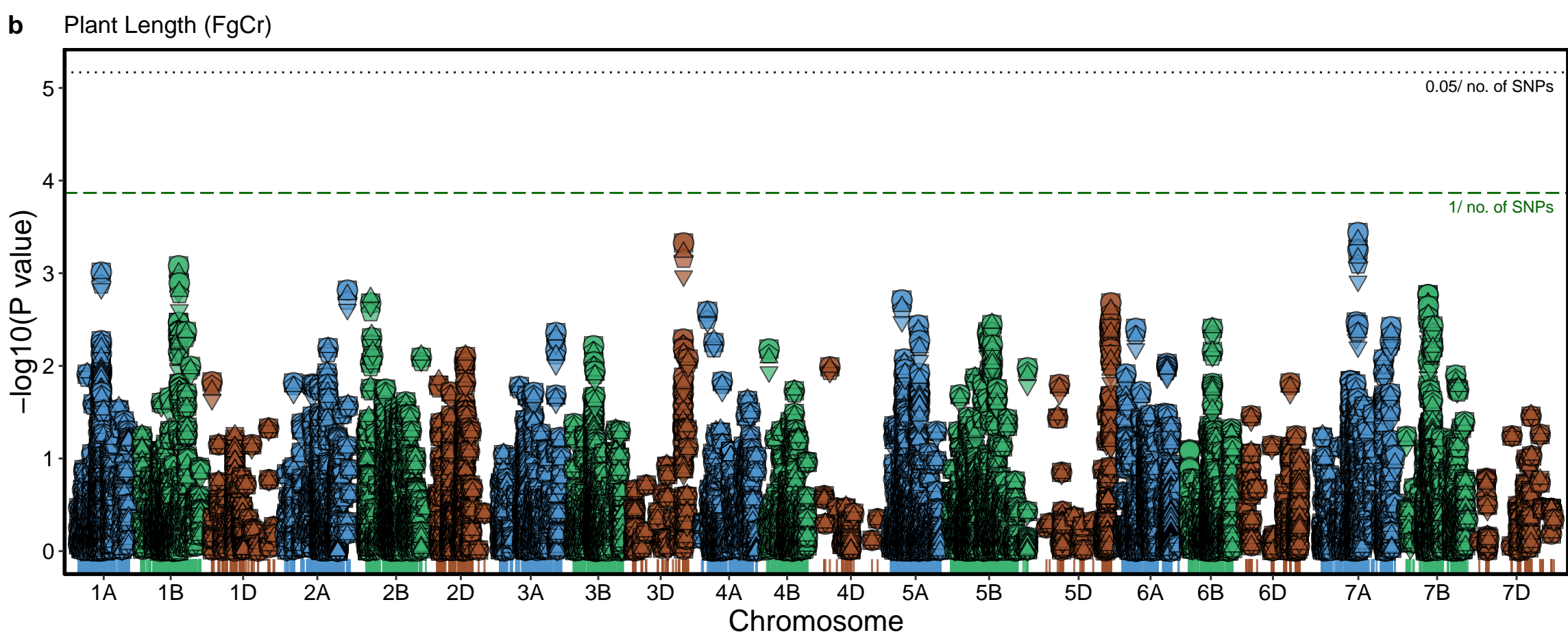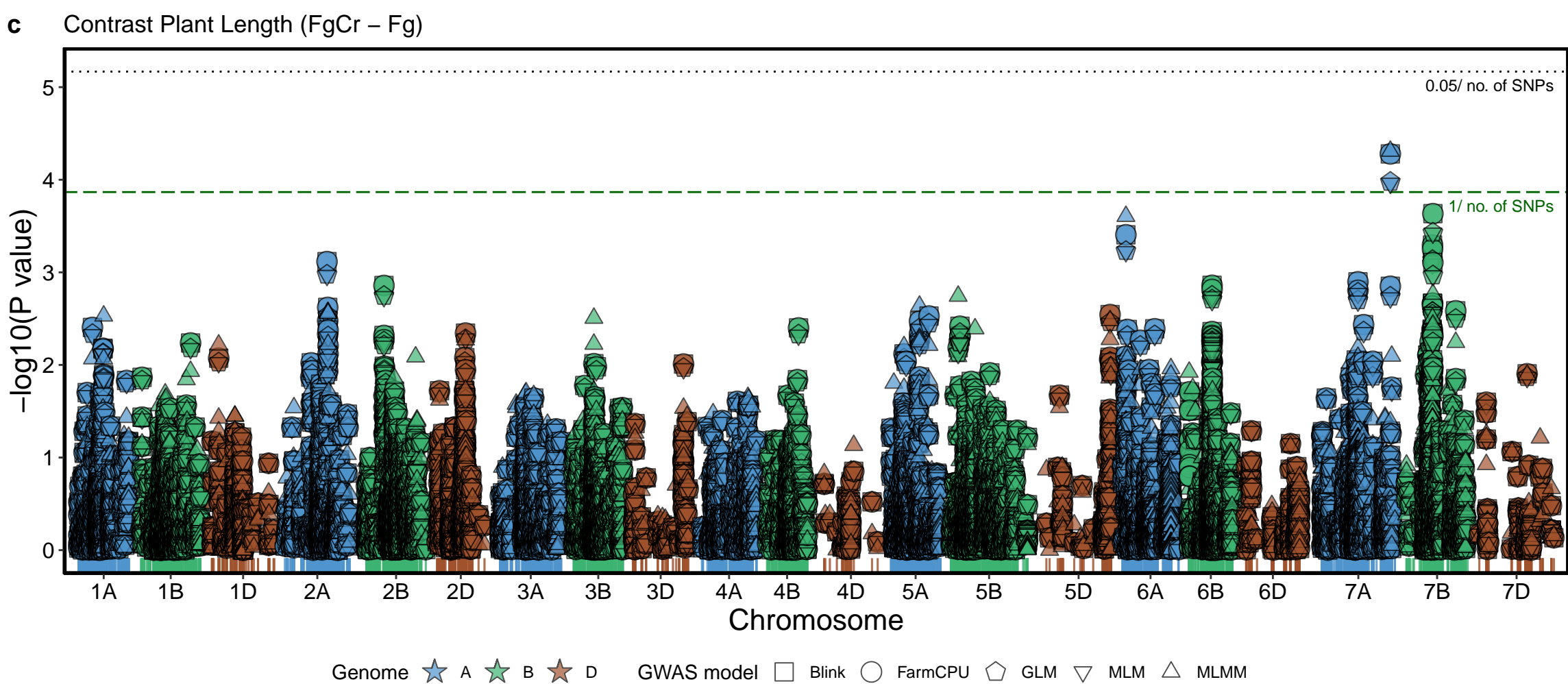

Supplement: jkae240_Supplementary_Data [file jkae240_supplementary_data.zip › Figure_S3_G3-2024-405205.pdf]

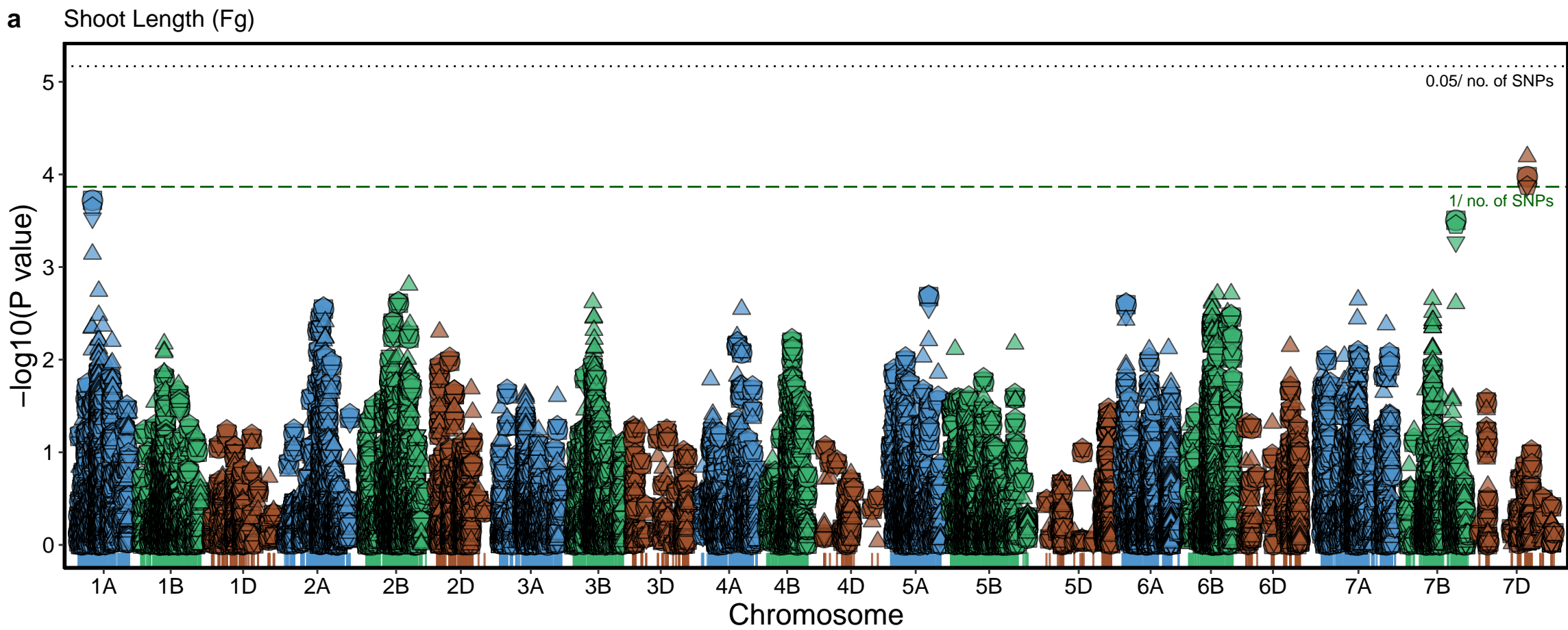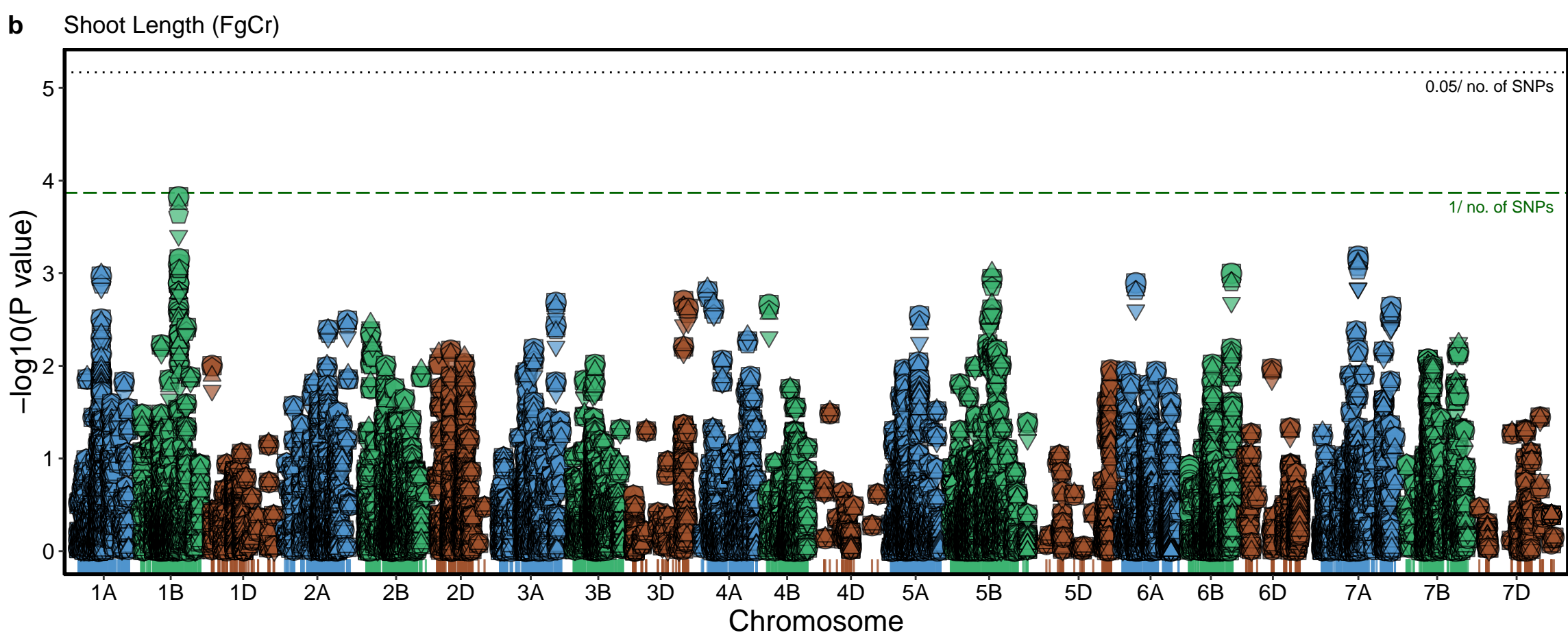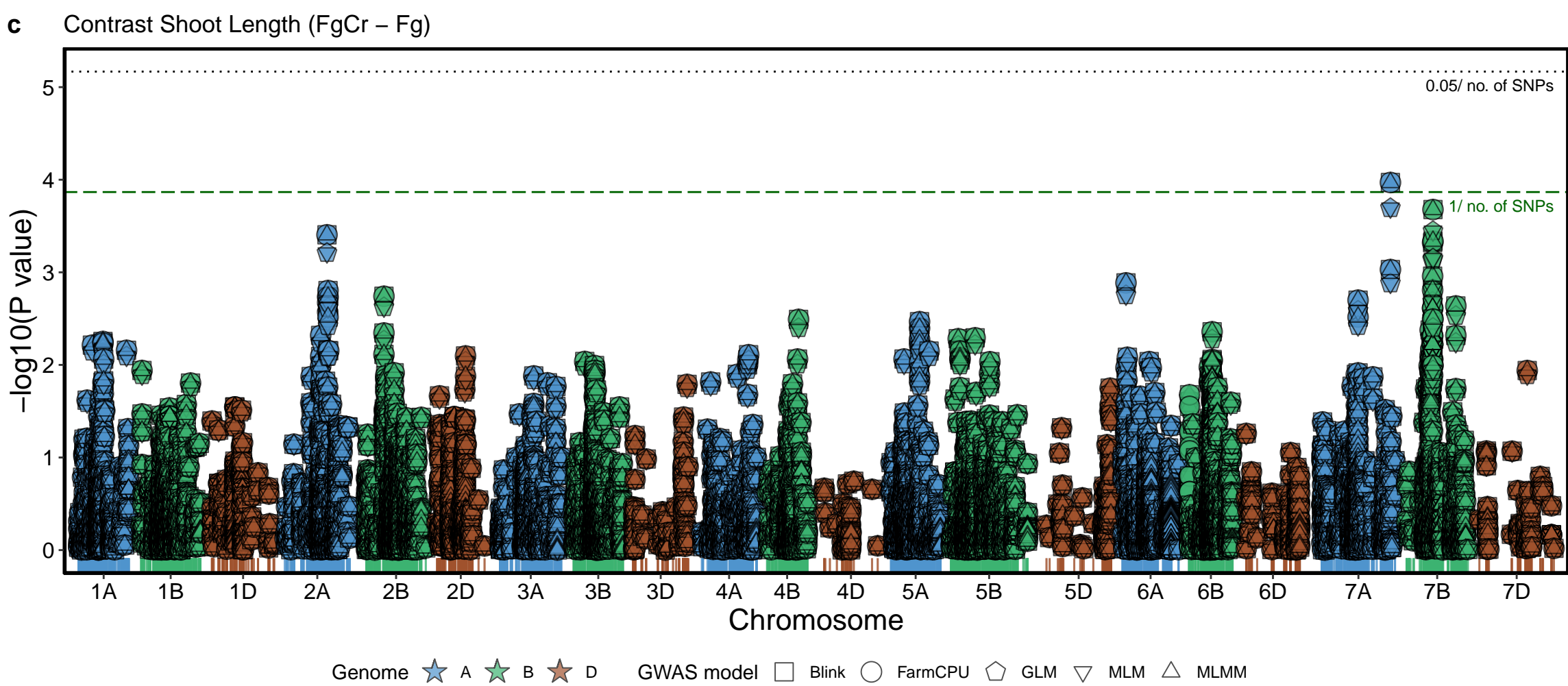

Supplement: jkae240_Supplementary_Data [file jkae240_supplementary_data.zip › Figure_S4_G3-2024-405205.pdf]

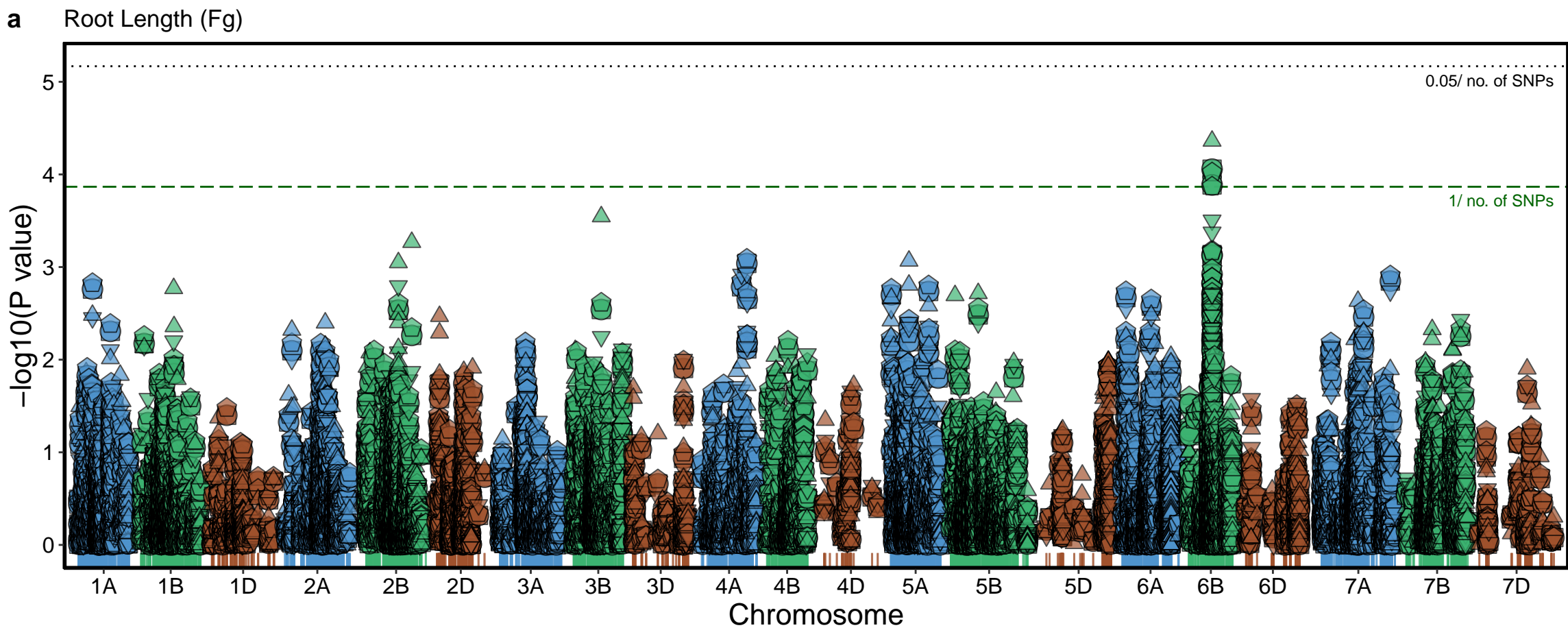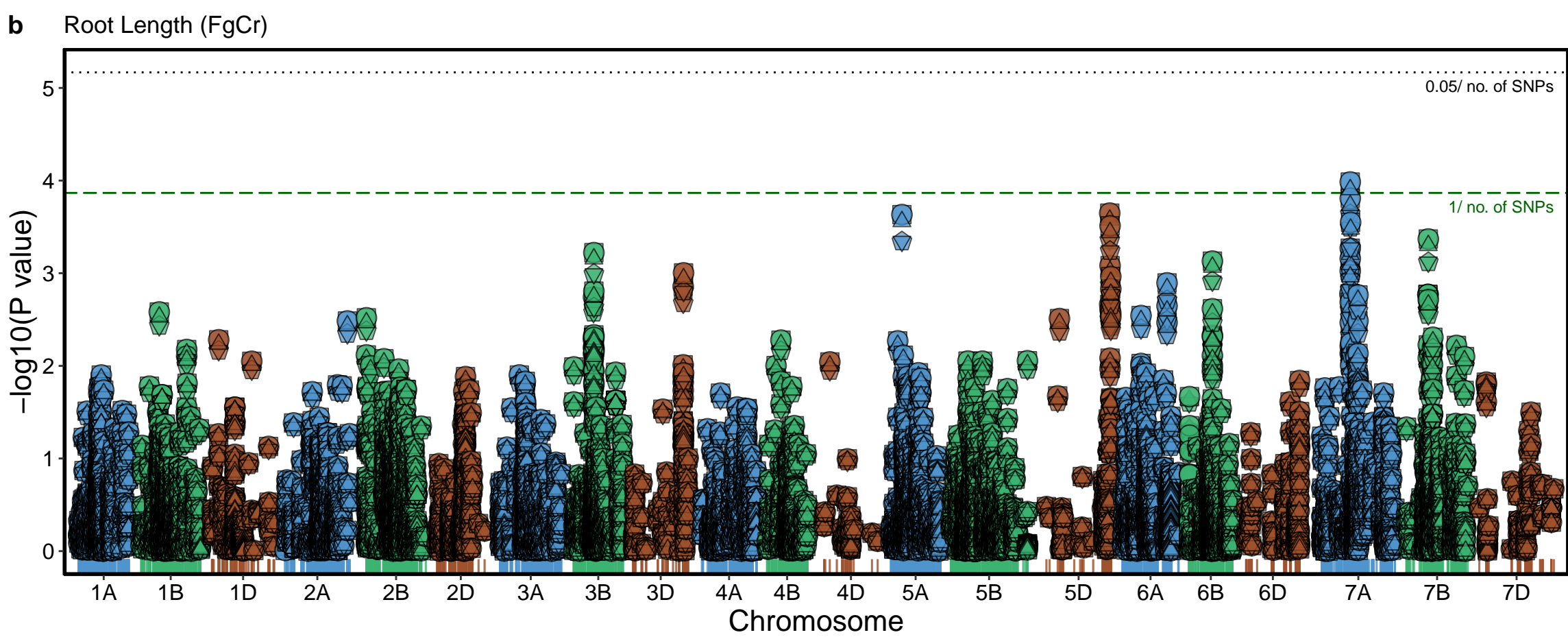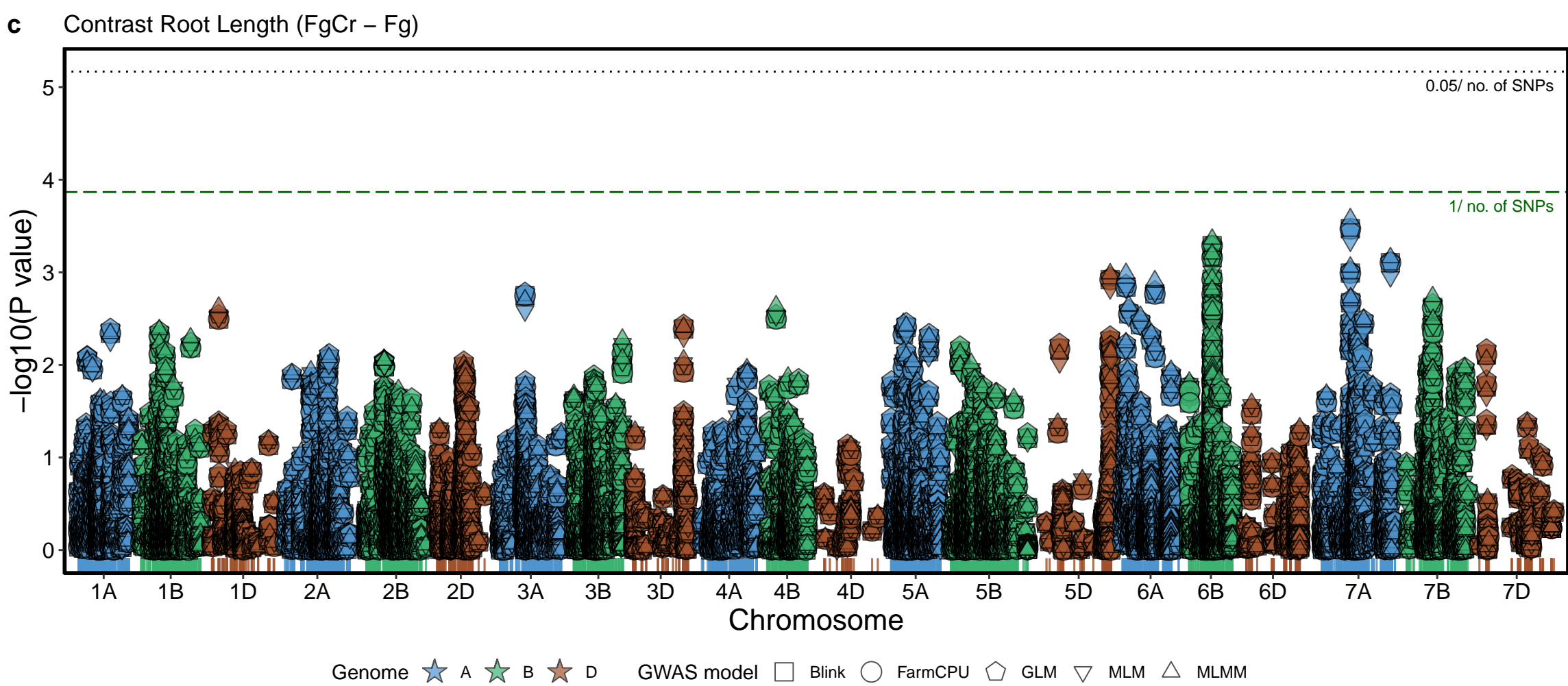

Supplement: jkae240_Supplementary_Data [file jkae240_supplementary_data.zip › Figure_S5_G3-2024-405205.pdf]
